# Computational Advances in Drug Safety: Systematic and Mapping Review of Knowledge Engineering Based Approaches

**DOI:** 10.3389/fphar.2019.00415

**Published:** 2019-05-17

**Authors:** Pantelis Natsiavas, Andigoni Malousi, Cédric Bousquet, Marie-Christine Jaulent, Vassilis Koutkias

**Affiliations:** ^1^Institute of Applied Biosciences, Centre for Research and Technology Hellas, Thessaloniki, Greece; ^2^Sorbonne Université, INSERM, Univ Paris 13, Laboratoire d'Informatique Médicale et d'Ingénierie des Connaissances pour la e-Santé, LIMICS, Paris, France; ^3^Laboratory of Biological Chemistry, Department of Medicine, Aristotle University of Thessaloniki, Thessaloniki, Greece; ^4^Public Health and Medical Information Unit, University Hospital of Saint-Etienne, Saint-Étienne, France

**Keywords:** drug safety, pharmacovigilance, knowledge engineering, knowledge discovery, knowledge representation, ontologies, terminologies, semantic technologies

## Abstract

Drug Safety (DS) is a domain with significant public health and social impact. Knowledge Engineering (KE) is the Computer Science discipline elaborating on methods and tools for developing “knowledge-intensive” systems, depending on a conceptual “knowledge” schema and some kind of “reasoning” process. The present systematic and mapping review aims to investigate KE-based approaches employed for DS and highlight the introduced added value as well as trends and possible gaps in the domain. Journal articles published between 2006 and 2017 were retrieved from PubMed/MEDLINE and Web of Science® (873 in total) and filtered based on a comprehensive set of inclusion/exclusion criteria. The 80 finally selected articles were reviewed on full-text, while the mapping process relied on a set of concrete criteria (concerning specific KE and DS core activities, special DS topics, employed data sources, reference ontologies/terminologies, and computational methods, etc.). The analysis results are publicly available as online interactive analytics graphs. The review clearly depicted increased use of KE approaches for DS. The collected data illustrate the use of KE for various DS aspects, such as Adverse Drug Event (ADE) information collection, detection, and assessment. Moreover, the quantified analysis of using KE for the respective DS core activities highlighted room for intensifying research on KE for ADE monitoring, prevention and reporting. Finally, the assessed use of the various data sources for DS special topics demonstrated extensive use of dominant data sources for DS surveillance, i.e., Spontaneous Reporting Systems, but also increasing interest in the use of emerging data sources, e.g., observational healthcare databases, biochemical/genetic databases, and social media. Various exemplar applications were identified with promising results, e.g., improvement in Adverse Drug Reaction (ADR) prediction, detection of drug interactions, and novel ADE profiles related with specific mechanisms of action, etc. Nevertheless, since the reviewed studies mostly concerned proof-of-concept implementations, more intense research is required to increase the maturity level that is necessary for KE approaches to reach routine DS practice. In conclusion, we argue that efficiently addressing DS data analytics and management challenges requires the introduction of high-throughput KE-based methods for effective knowledge discovery and management, resulting ultimately, in the establishment of a continuous learning DS system.

## Introduction

Pharmacovigilance (PV)^1^, also known as Drug Safety (DS), is “*the science and activities related to the detection, assessment, understanding and prevention of adverse effects or any other possible drug-related problems*” (World Health Organization, 2002). DS is an important issue of public health interest, given that adverse drug reactions (ADRs^2^) and adverse drug events (ADEs^1^) cause a significant social and financial burden^3^. An important part of DS concerns the identification of the so-called “signals”^4^, performed by national and international drug monitoring/regulatory organizations (e.g., the Uppsala Monitoring Centre (UMC), the European Medicines Agency (EMA), the Food and Drug Administration (FDA) in the United States, etc.). Signal detection is typically based on the analysis of individual case safety reports gathered in Spontaneous Reporting Systems (SRSs), e.g., using disproportionality-based statistical methods (Montastruc et al., 2011).

The current era of “data explosion” affects the entire spectrum of health, including DS. While traditionally post-marketing DS surveillance relied on SRSs as well as clinical studies and the scientific literature, advances in Information and Communication Technologies (ICT) recently enabled the exploitation of new/emerging data sources, such as observational healthcare databases, biochemical and genetic databases, social media, internet search logs, etc. To this end, various computational analysis methods have been proposed for post-marketing DS surveillance (Harpaz et al., 2012), illustrating both strengths and weaknesses (Hauben and Norén, 2010).

For the development and safety monitoring of new drugs (i.e., prior to market authorization), computational approaches attract lately a major interest as well, especially in the scope of *in silico clinical trials* (Pappalardo et al., 2018) and *Precision Medicine* (Collins and Varmus, 2015). Multi-scale modeling approaches (exploiting low-level biochemical information regarding the behavior of molecular structures as well as more abstract information regarding the phenotypic action of a drug via mathematic models, systems, or network-based structures) are being used in *Systems Pharmacology* (SP) (Mager and Kimko, 2016). In particular, SP-based approaches have been used for DS (Bai et al., 2014; Boland et al., 2016; Schotland et al., 2016; Trame et al., 2016) and regulatory actions (Lorberbaum et al., 2015), as they facilitate *in silico clinical trials* (Ramanujan et al., 2016; Rieger et al., 2018), including the simulation of individual patient characteristics toward the overall vision of *Precision Medicine* (Birtwistle et al., 2016).

To this end, the recent data deluge dictates the need to introduce high-throughput computational methods for DS that will enable efficient knowledge extraction and management, compensating the underlying data heterogeneity and complexity. This need becomes more demanding, especially considering the concurrent investigation of diverse types of data, in order to strengthen the evidence of the outcomes provided by the respective computational methods (Koutkias and Jaulent, 2015).

In Computer Science, *knowledge* is represented “by facts, rules and other symbolic structures, rather than the traditional representation as abstract numbers or algorithms” (Fox, 1984). *Knowledge Engineering* (KE) is the discipline that elaborates on the theories, methods, and tools for developing knowledge-intensive applications (Schreiber, 2008). KE typically entails: (a) *knowledge extraction* (e.g., based on Natural Language Processing (NLP)^5^), (b) *knowledge integration* (i.e., syntactic and semantic alignment as well as normalization of different kinds of knowledge), (c) *knowledge representation* (i.e., modeling of domain/application knowledge in computationally exploitable formats like ontologies^6^), (d) *knowledge dissemination* (i.e., modeling information for communication purposes focusing for example on interoperability among heterogeneous ICT systems), and (e) *knowledge elicitation* (i.e., generating or discovering new knowledge via advanced KE techniques like semantic mining).

Recent research has illustrated that KE can contribute in addressing DS challenges. In particular, KE applications for DS can facilitate the integration and analysis of heterogeneous data sources (Koutkias and Jaulent, 2015), and represent the respective knowledge in a manner which facilitates advanced processing capabilities like automatic inference (Natsiavas et al., 2018). The later requires the definition of explicit semantics via well-defined knowledge structures, i.e., common reference terminologies, thesauri, or ontologies. The use of such reference knowledge structures is a key aspect in KE, as it facilitates “machine-understandable” interlinking, comparison, reuse and further processing of data in two ways: (a) it enhances semantic interoperability through common reference concepts, and (b) it provides the underlying semantic infrastructure for automatic inference. Thus, the use of reference knowledge structures is crucial in order to characterize a computational method/system as “knowledge-based.”

In KE, semantics are expressed via relationships among the referred concepts (e.g., “Myocardial Infarction” *occurs_in* “Myocardium”), or via a hierarchy of concepts and their properties using “*sub-concepts*” (e.g., the term “Myocardial Infarction” *is_a* “Cardiac Disorder”) and “*sub-properties*,” respectively. A knowledge structure could describe how ADEs, such as “myocardial infarction” may be associated to the corresponding pathological process and anatomical location, e.g., “Myocardial Infarction” *is_a* “Cardiac Disorder” *and occurs_in* “Myocardium.” An ICT system would represent this knowledge and the respective concepts using a reference terminology, e.g., MedDRA^7^. Such an explicit and computationally exploitable representation of knowledge enables “reasoning.” As an example of how a computer may perform automatic reasoning, explicit linking of an ADE to its corresponding biological process (e.g., “Cardiac Failure” *is_associated_with* “Heart Contraction”) allowed the identification of 190 genes that are associated with heart contraction and could potentially have a role in cardiac failure (Sarntivijai et al., 2016).

This study constitutes a “systematic and mapping review”^8^, conducted in accordance with the Preferred Reporting Items for Systematic Reviews and Meta-Analyses (PRISMA) statement (Moher et al., 2009). It aims to present KE-based approaches for DS and their potential application in current DS practice, illustrating the added value through exemplar research efforts spanning diverse dimensions of DS research. Thus, the main research question of the current study is: “*What are the main KE methods applied in the DS domain, upon which knowledge models and data sources are they applied, what is their contribution/added value for DS, and what are the potential gaps, challenges and opportunities for further research?*”.

## Methods

**Figure 1:**
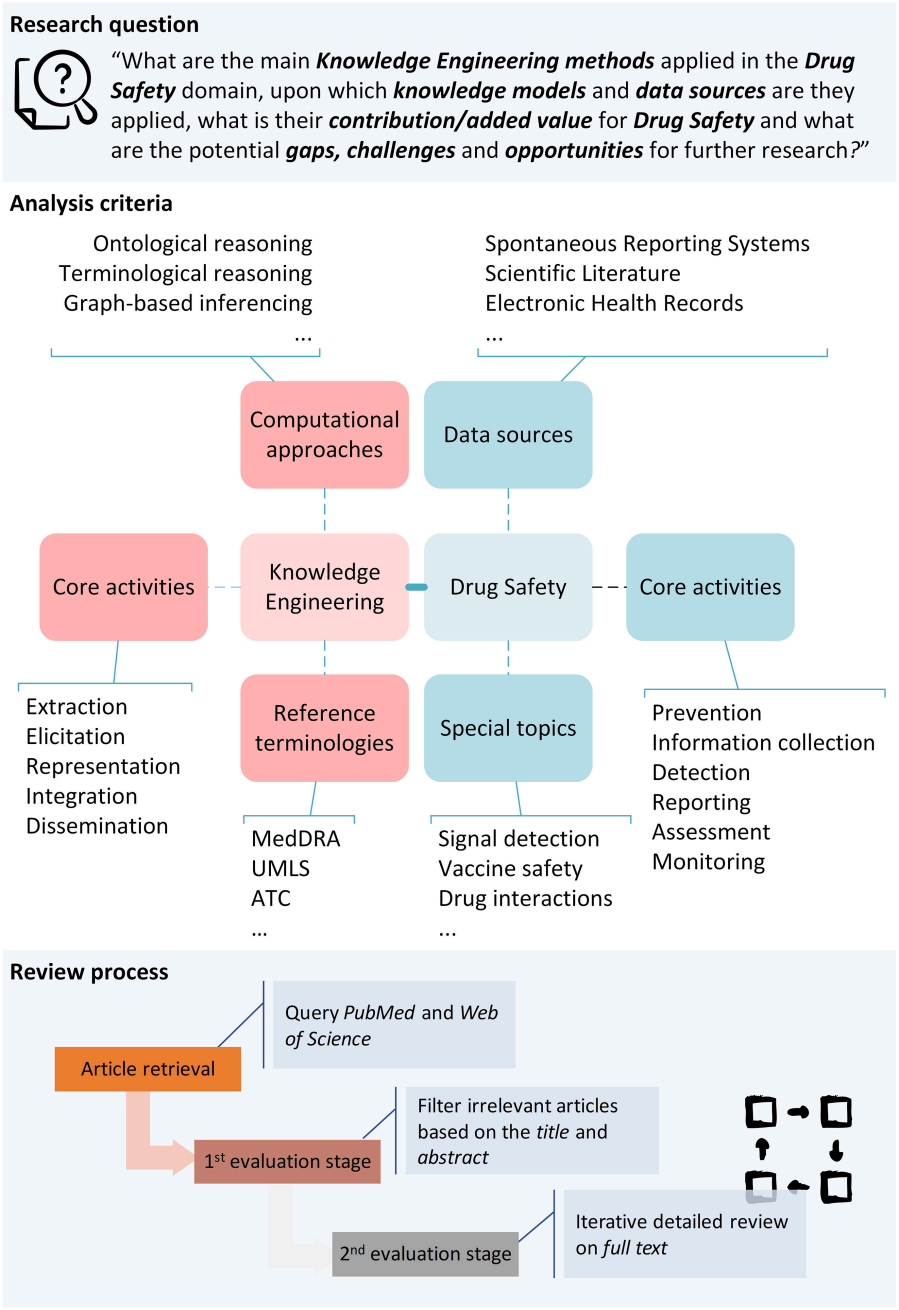
Rationale of the review methodology.

A systematic search was performed by querying two reference bibliographic repositories: PubMed^9^ and Web of Science^10^. The study comprised of the article retrieval step and two consecutive review stages (Figure 1); the first aimed to filter irrelevant articles with the domains of KE and DS based on their title and abstract, and the second was devoted to evaluating the remaining papers' full-text in detail, and map them based on specific analysis criteria.

The review was conducted by the authors of the paper. In the retrieval stage, we defined and executed two queries (provided as Supplementary Material) and imported the obtained citations in BibReview^11^, a tool that was used throughout our study enabling collaborative review of bibliographic data (Lamy et al., 2015). The queries included two core parts (linked with the logical operator AND), each comprising of synonym terms describing the domains of interest, i.e., KE and DS. We considered articles written in English and published in scientific journals between 2006 and 2017. 2006 was selected as the starting year of our review, since there was no much activity on KE for DS until then and a key study regarding the use of MedDRA has sparkled an interesting discussion about the use of formal semantics, highlighting the prospects and the need for further research (Bousquet et al., 2005b).

In addition, the current study relied on the following inclusion and exclusion criteria:

1. Inclusion criteria: (a) articles exploiting clearly KE methods/technologies; (b) articles referring to algorithms exploiting formal mathematic structures (e.g., graphs), as these can be considered knowledge representation schemes, and (c) articles in which NLP was employed to extract information from free-text sources combined with other KE processes, e.g., ML algorithms using reference terminologies/ontologies.
2. Exclusion criteria: (a) articles referring to “inference” based on plain statistics; (b) articles referring to ontologies [e.g., Gene Ontology (GO)] as simple data sources, without exploiting their underlying semantics; (c) articles not reporting the use of at least one knowledge source, e.g., a terminology, a thesaurus, an ontology, etc.; (d) opinion or review papers not providing concrete suggestions or designs, and (e) articles from the same authors with a high degree of overlapping^12^.

Table 1 partially presents the analysis criteria employed in the overall mapping process. These were based on established knowledge in the domain, experiences and tacit knowledge of the authors, and the outcomes obtained as the review progressed. While other systematic reviews related with KE were considered for criteria definition (e.g., Bjørnson and Dingsøyr, 2008; Wnuk and Garrepalli, 2018), to a great extent these were found irrelevant for our study. In order to reduce the subjectivity of the review process, specific enumerations of answers for each review criterion were defined. The authors iteratively examined the possible answers for each criterion, to make sure that these are orthogonal (not conceptually overlapping) to the extent possible. Furthermore, specific explanations for each criterion value were added in a spreadsheet file used for data gathering and analysis^13^, in order to avoid ambiguities for the reviewers.

**Table 1:** Analysis criteria and indicative answers.

**Criterion**	**Indicative answers**
DS core activities	ADE information collection, ADE detection, ADE assessment, ADE monitoring, ADE prevention, ADE reporting
DS special topics	Comparative drug analysis, Drug interactions, MoA identification/analysis Personalized drug safety, Signal detection, Specific (class of) disease, Specific (class of) drug(s), Specific adverse effect, Vaccine safety
Data source categories	ADE databases, Bibliographic databases, Clinical narratives, Clinical trials, Drug information databases, EHRs, Genetics and biochemical databases, HL7 messages, Manually annotated corpora, mHealth apps, Patient summaries, PHRs, Social media, Structured Product Labels, Spontaneous Reporting Systems
Data source(s)	Absorption, Distribution, Metabolism, and Excretion Associated Proteins database (ADME-APs), ADE Corpus, ADEpedia, ADRMine Corpus, AEOLUS, AERS-DM, etc.
KE core activities	Knowledge dissemination, Knowledge elicitation, Knowledge extraction, Knowledge integration, and Knowledge representation
Computational method(s)	Data mining, Disproportionality analysis, Graph-based inferencing, Information extraction (e.g., Natural Language Processing), Machine Learning, Ontology reasoning, Rule-based inferencing, Simulation, Terminological reasoning, Vector-based similarity identification
Challenges/weaknesses	Commercial tools, Competing interests, Evaluation against small dataset, Evaluation restricted on a narrow scope, Evaluation with simulated data, Knowledge model not available, Knowledge model not validated for completeness, No evaluation regarding knowledge modeling quality criteria, No statement regarding competing interests, Not applying formal DL semantics, Not using a knowledge representation standard, Proprietary datasets, Significant dependence on manual work
Reference terminologies/ontologies	Adverse Event Reporting Ontology (AERO), Anatomical Therapeutic Chemical (ATC) classification system, Basic Formal Ontology (BFO), British National Formulary (BNF) Dictionary, ChEBI, etc.
Knowledge formalism	DAML + OIL, Frame-based ontology, OWL, RDF, Relational, SWRL, XML
Country	E.g., Australia, Belgium, Canada, China, Denmark, France, etc.
Organization type	Academia/Research, Healthcare, Industry, DS Monitoring

In order to mitigate the risk for various kinds of bias, we applied the guidelines provided by Drucker et al. (2016) and Altman et al. (2011), which are further discussed in subsection Risk of Bias.

## Results

The analysis results are provided as Supplementary Material in the form of a spreadsheet, while they are also publicly available as online interactive analytics forms, enabling their investigation in further detail^14^. This section presents the most important facets of these results.

### Article Selection

**Figure 2:**
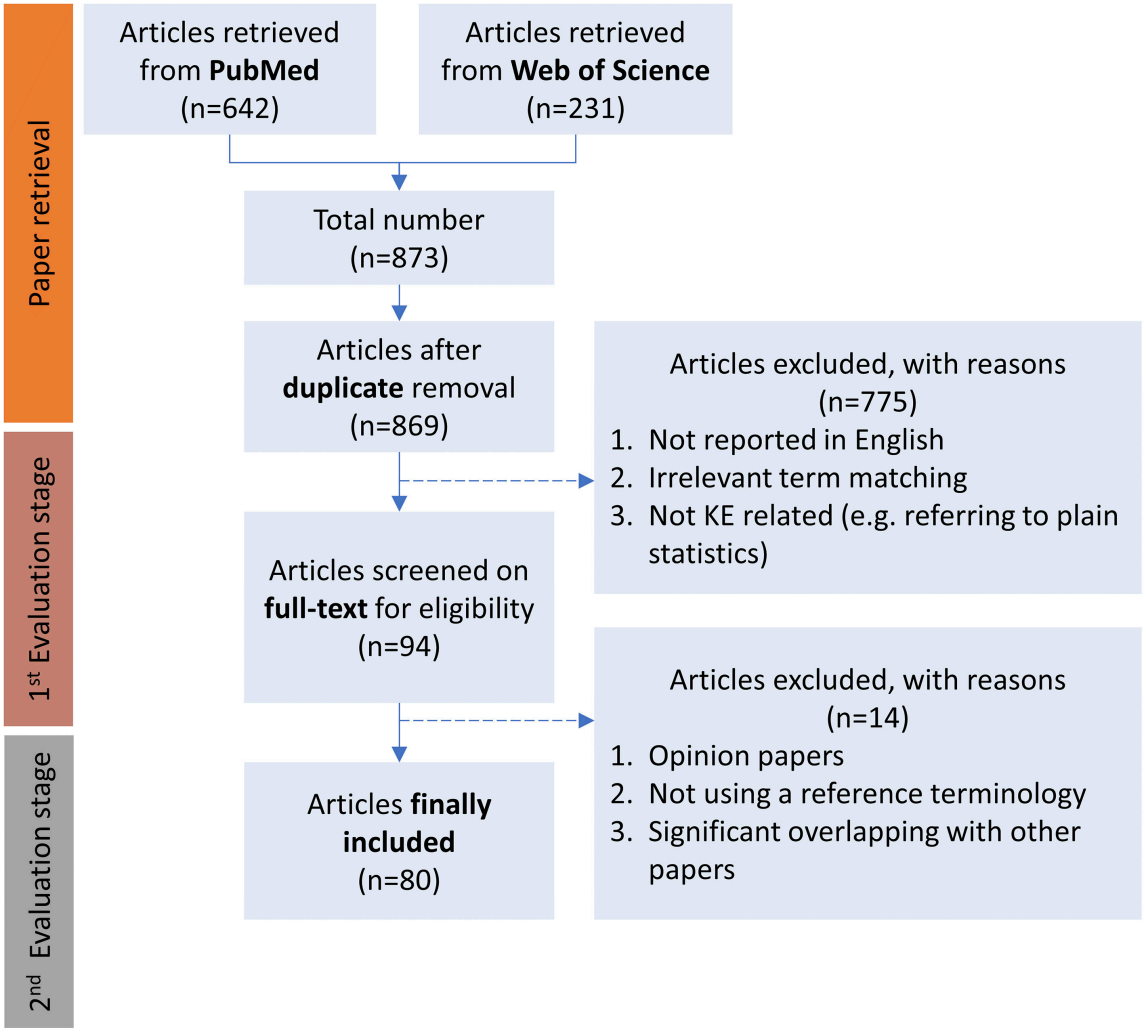
The PRISMA flow in the context of the current study.

Figure 2 depicts the number of selected papers in each step of the review process, following the PRISMA guidelines (Moher et al., 2009). From the 873 articles initially retrieved, 94 articles were selected to be evaluated in full detail. 14 of them were excluded during the full-text review according to the exclusion criteria defined (section Methods). Finally, 80^15^ articles were included in the presented review.

**Figure 3:**
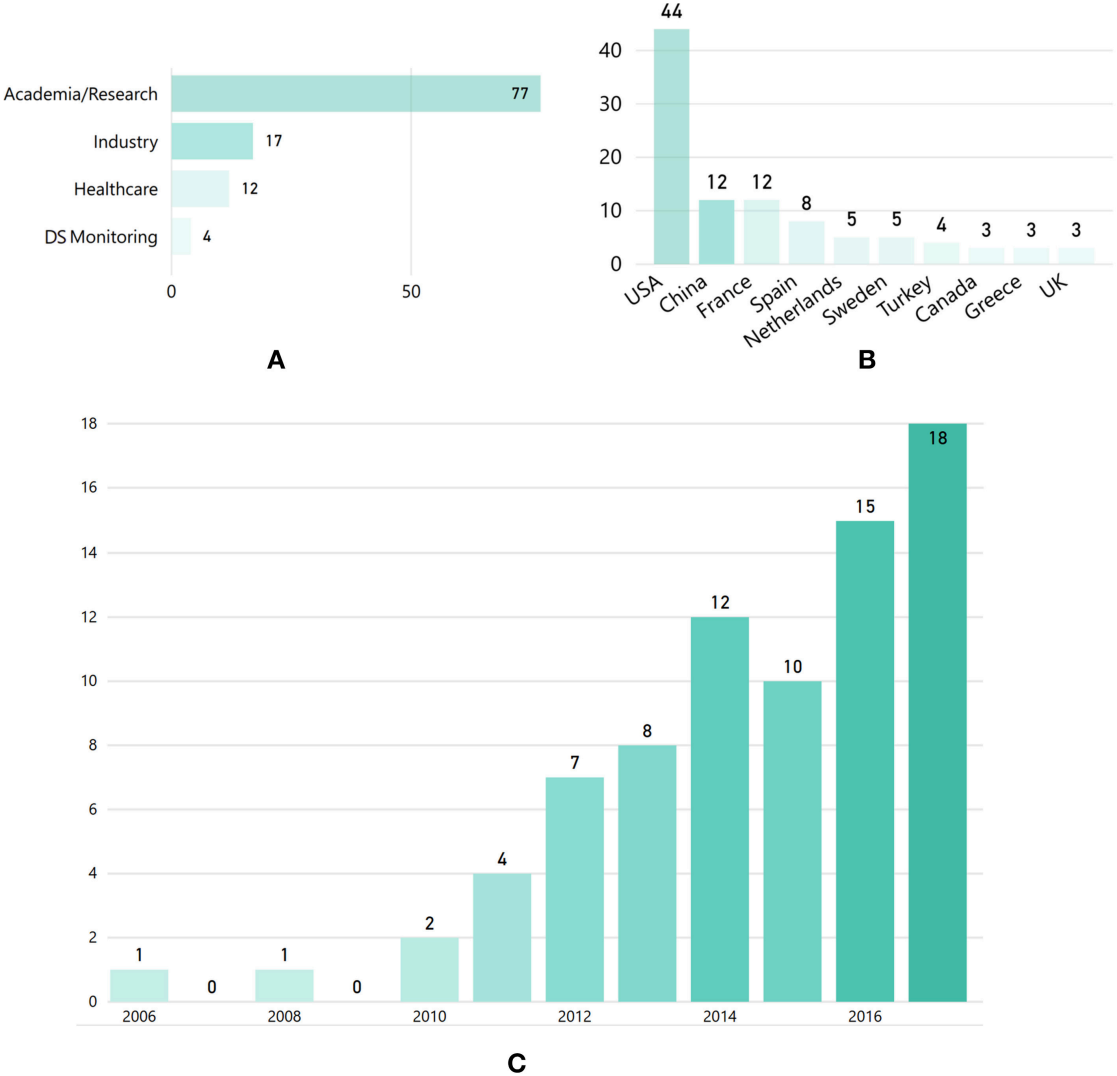
**(A)** Number of articles per authors' organization category, **(B)** author-country distribution (showing only *n* > 3 articles), and **(C)** distribution of the selected articles per year.

The “demographic” features of the selected articles are illustrated in Figure 3. In particular, Figure 3A presents the distribution of articles according to the organization category of the respective authors, highlighting that industrial, healthcare and DS monitoring organizations contributed less in the domain, compared to research organizations. As shown in the author-country distribution depicted in Figure 3B, most articles were produced by organizations located in the USA. However, China, France and Spain are also among the leading countries in researching KE for DS. In terms of time evolution, Figure 3C depicts an increasing trend in the number of publications after 2010.

### Synthesized Findings

In this section, we present in detail the results of our quantified analysis based on the criteria presented in Table 1. Furthermore, we provide an overview of the impact of the selected papers on the main topics posed by the study research question, as described in the Introduction section^16^.

#### Quantified Analysis

**Figure 4:**
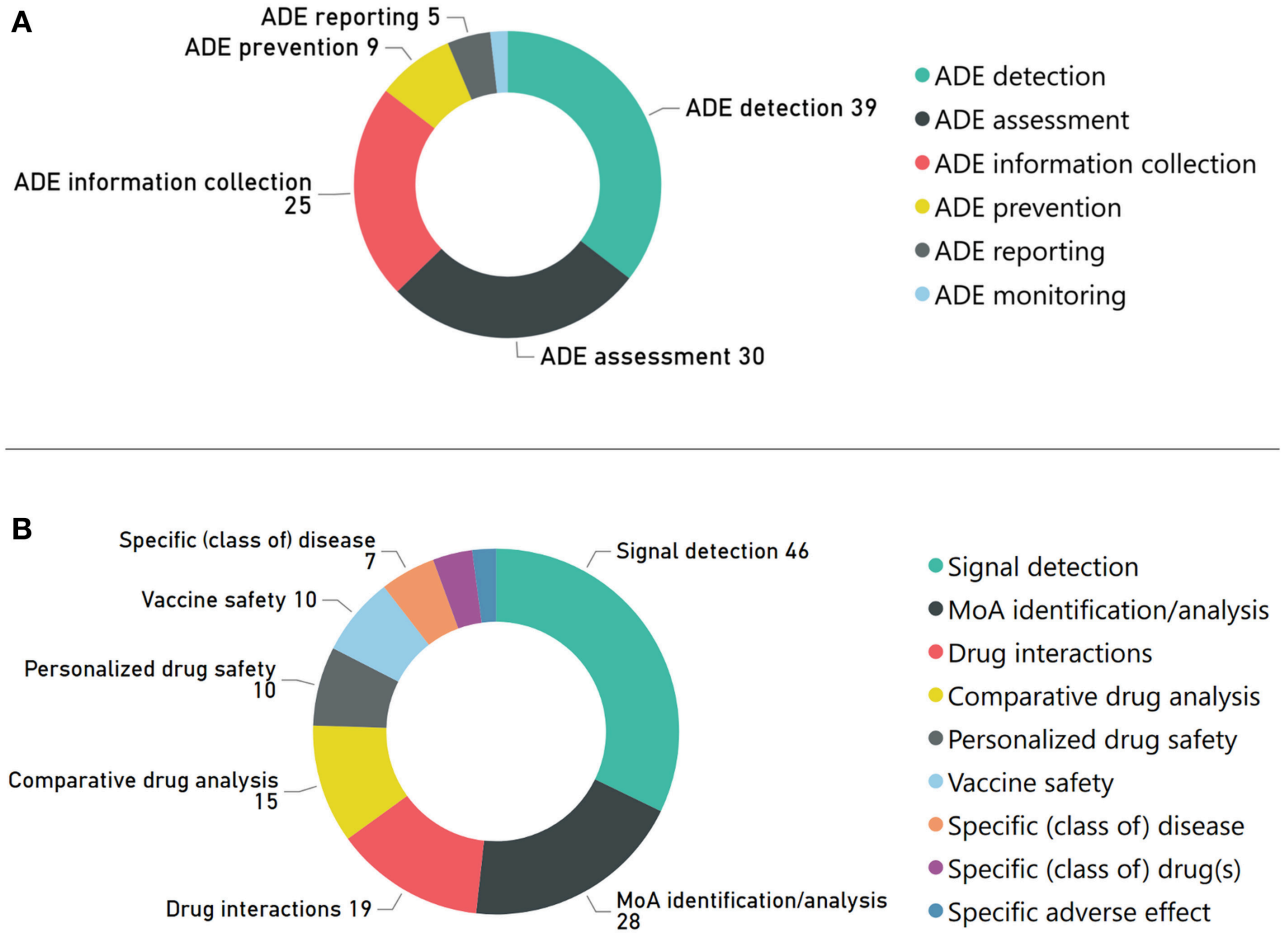
Number of articles related with: **(A)** DS core activities, and **(B)** DS special topics.

Figure 4 depicts the distribution of the reviewed articles, according to the DS core activities and special topics. As shown in Figure 4A, “ADE detection-”, “ADE information collection”, and “ADE assessment” attract most research efforts among the core DS activities. Respectively, Figure 4B depicts that signal detection, mechanism of action (MoA) analysis, and drug interactions are the leading DS special topics.

**Figure 5:**
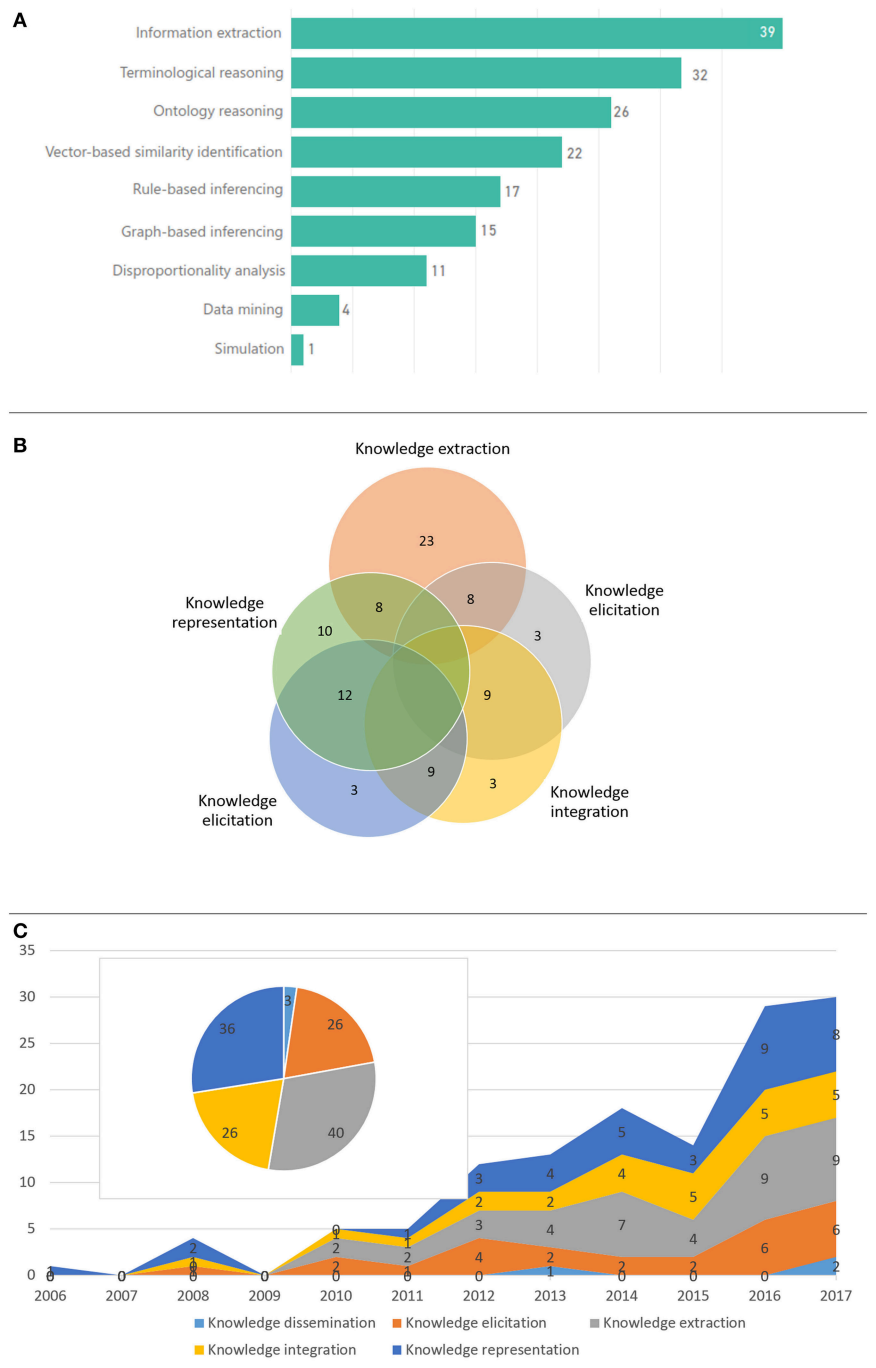
KE and computational approaches: **(A)** number of articles per computational approach, **(B)** overlapping of the most prominent KE activities within the selected articles, and **(C)** KE activities and number of respective articles across time.

Figure 5 depicts the main KE activities employed in the reviewed articles and their time evolution (Figure 5C), as well as the number of articles related with the most prominent computational approaches (Figure 5A). Knowledge extraction, representation and elicitation were the main focus, mostly through the application of NLP, terminological reasoning, ontological reasoning and vector-based similarity identification using ML algorithms, e.g., Support Vector Machines (SVMs). Typically, more than one KE core activities were employed in each article. As shown in Figure 5B only knowledge extraction seems to be a standalone approach, which has been employed in a significant number of papers. This use of more than one KE core activities outlines the complexity of the targeted problems and the need for synthesized approaches to address them.

**Figure 6:**
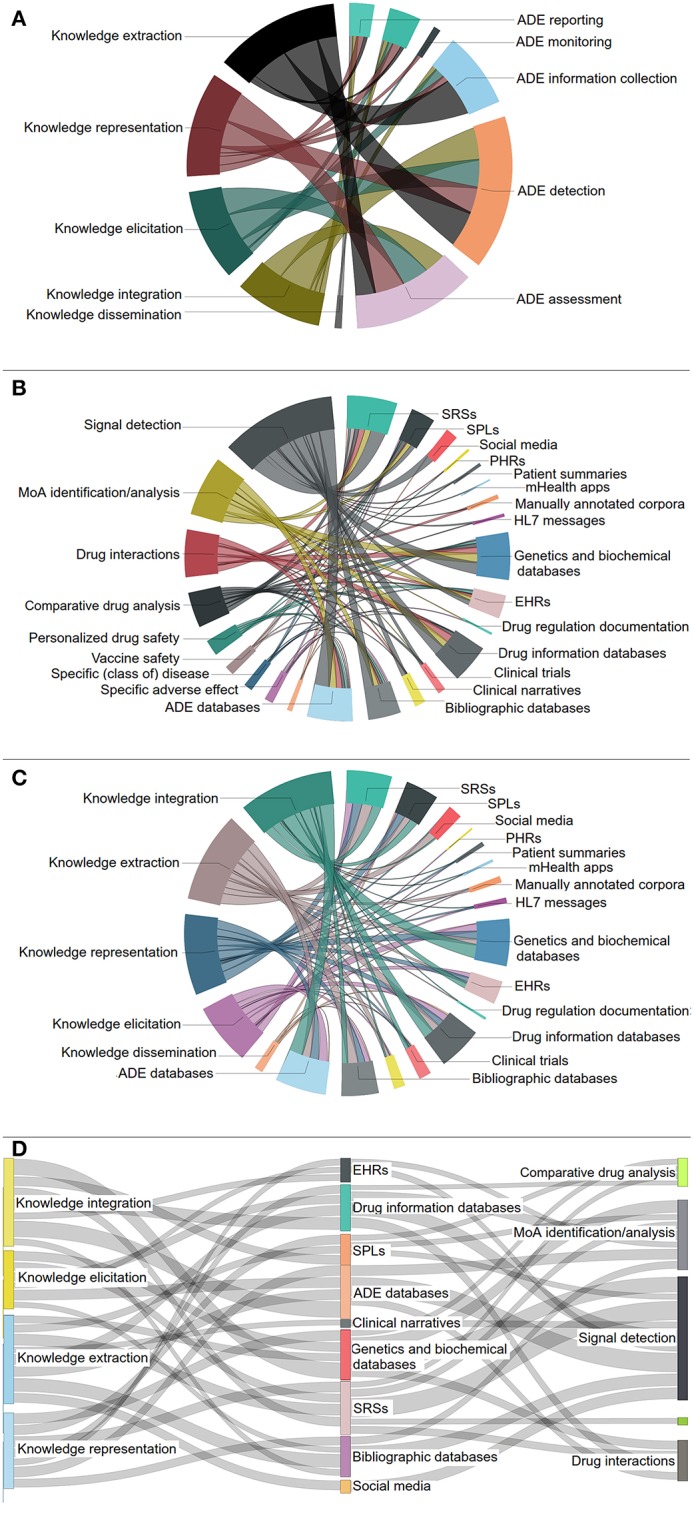
Links between: **(A)** KE core activities and DS core activities, **(B)** DS special topics and data source categories, **(C)** KE core activities and data source categories. **(D)** The most prominent connections among KE core activities, data source categories and DS special topics.

Figures 6A–C present the associations between the various DS and KE core activities, the DS special topics and the data source categories, as well as the KE core activities and the data sources, respectively, in the form of chord diagrams. Figure 6D depicts a Sankey diagram presenting the most significant interconnections^17^ among the DS special topics, the most important data source categories, and the KE core activities based on the reviewed articles. Interestingly, “signal detection,” “MoA analysis and identification,” and “Drug interactions” are the three most elaborated DS special topics, exploiting a number of heterogeneous data sources, e.g., SRSs, ADE databases, etc.

**Figure 7:**
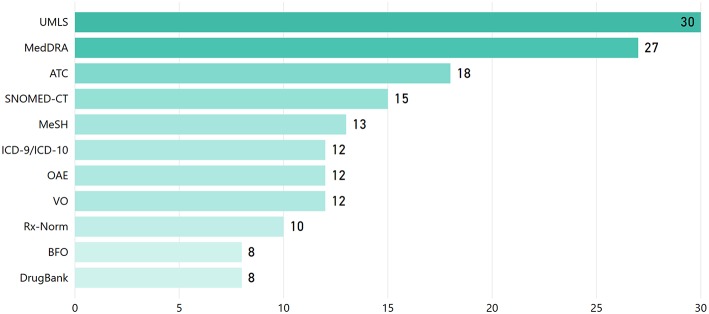
Reference knowledge sources (i.e., terminologies/vocabularies/thesauri and ontologies) employed in the reviewed articles.

One of the key KE foundations is the reuse of established/reference knowledge structures (i.e., ontologies, standard terminologies, etc.). This facilitates semantic interoperability between different systems and widens the spectrum upon which KE approaches are applicable to. Figure 7 presents the most widely adopted terminologies/ontologies in the reviewed articles^18^. The Unified Medical Language System (UMLS), MedDRA, the Anatomical Therapeutic Chemical (ATC) Classification System, the Systematized Nomenclature of Medicine-Clinical Terms (SNOMED-CT), and the International Classification of Diseases (ICD) are the most widely used terminologies, while the Ontology for Adverse Events (OAE), the Vaccine Ontology (VO), and GO are the most widely referred ontologies.

**Figure 8:**
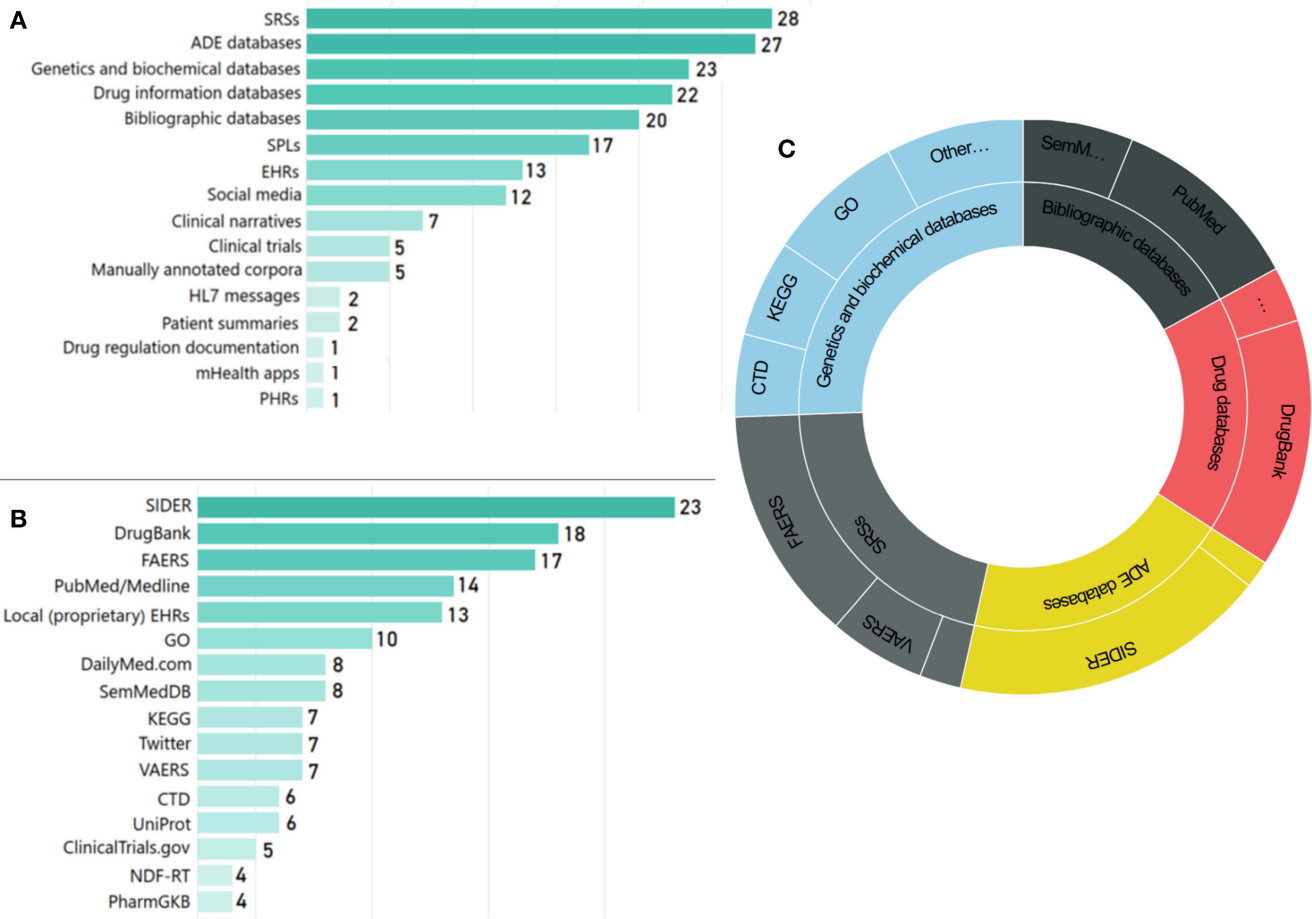
Use of main data sources: **(A)** number of articles per data source category, **(B)** number of articles per data source, and **(C)** schematic representation of main data sources used and their categories.

The types of data sources employed in the reviewed articles vary significantly, highlighting the complexity of the domain and the need for advanced data integration and representation schemes based on KE (Koutkias and Jaulent, 2015). Figure 8A presents the distribution of data source categories, while Figure 8B presents the most popular data sources^19^, as employed in the reviewed articles. SRSs [e.g., the FDA Adverse Event Reporting System (FAERS) and the Vaccine Adverse Event Reporting System (VAERS)], drug information databases (e.g., DrugBank), ADE databases [mainly the Side Effect Resource (SIDER)], genetic and biochemical information data sources [e.g., GO and the Kyoto Encyclopedia of Genes and Genomes—GenomeNet (KEGG)], as well as scientific literature repositories (i.e., PubMed/MEDLINE) are the most prominent ones.

**Figure 9:**
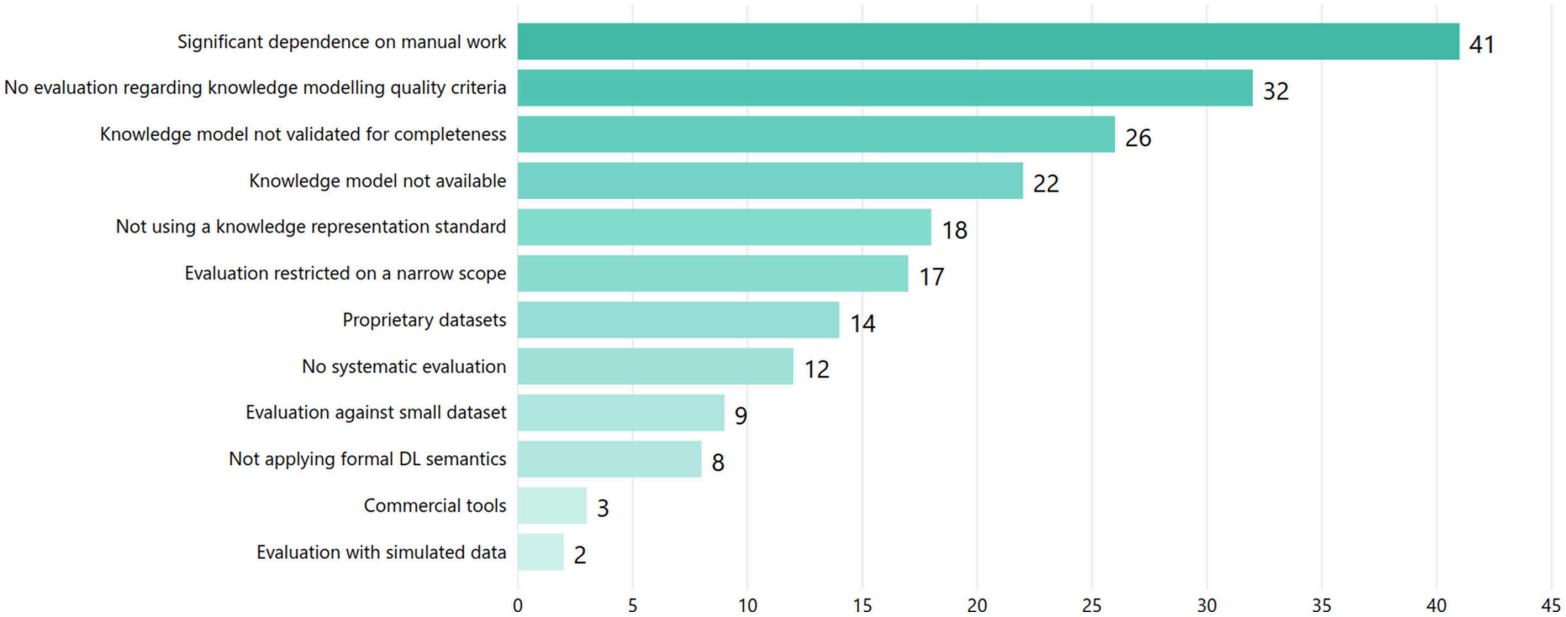
Identified challenges/weaknesses as reported in the reviewed articles.

The selected articles were also critically reviewed to identify challenges or weaknesses and, consequently, gaps in the applied research practices. As shown in Figure 9, in many of the reviewed articles the research significantly depended on manual work (e.g., data curation, annotation, etc.), conducted by a small group of experts. Furthermore, despite elaborating on KE representation schemes like ontologies, many studies did not evaluate the proposed models regarding quality, e.g., using quality assessment frameworks like the Ontology Quality Evaluation Framework (OQuaRE) (Duque-Ramos et al., 2014). This finding may indicate a difficulty to apply the respective approaches at large-scale with real-world data. Moreover, a wide range of studies did not use an interoperable knowledge representation format (e.g., ontologies), while in many studies the presented KE approaches were evaluated in a narrower scope than the one presented as their main use case.

#### Data and Knowledge Sources

In this subsection, we present the main data sources used in KE for DS, as well as the employed knowledge sources, i.e., reference ontologies/terminologies, as identified in our review^20^.

##### Data sources

Table 2 presents the usage of data sources for specific DS applications, citing also the respective articles. We organize data sources in two main types: (a) those *established* or dominant in the domain of DS, such as SRSs, clinical trial databases, and bibliographic databases, and (b) *emerging* or quite new, such as observational healthcare databases, biochemical/genetic information databases, and social media platforms.

**Table 2:** Use of data sources in the reviewed articles for most prominent DS applications.

	**Category**	**Application in drug safety**
Established data sources	SRS	*Signal detection* (Sarntivijai et al., 2012; Tao et al., 2012; Cheng et al., 2013; Boyce et al., 2014; Cheng and Zhao, 2014; Courtot et al., 2014; Iyer et al., 2014; Wang et al., 2014; Cai et al., 2015, 2017; Dupuch and Grabar, 2015; Liu and Chen, 2015; Koutkias and Jaulent, 2016; Liu et al., 2016, 2018; Knowledge Base Workgroup of the Observational Health Data Sciences and Informatics (OHDSI) Collaborative, 2017; Voss et al., 2017) *Validation* (Henegar et al., 2006; Gottlieb et al., 2012; Courtot et al., 2014; Iyer et al., 2014) *Monitoring* (Marcos et al., 2013)
	ADE databases	*Signal detection* (Huang et al., 2011; Gurulingappa et al., 2012; Cheng et al., 2013; Cheng and Zhao, 2014; Iyer et al., 2014; Shang et al., 2014; Cai et al., 2015; Herrero-Zazo et al., 2015; Jiang et al., 2015; Koutkias and Jaulent, 2015, 2016; Bravo et al., 2016; Eshleman and Singh, 2016; Kawazoe et al., 2016; Lowe et al., 2016; Noor et al., 2016; Abdelaziz et al., 2017; Knowledge Base Workgroup of the Observational Health Data Sciences and Informatics (OHDSI) Collaborative, 2017; Nguyen et al., 2017) *MoA identification/analysis* (Huang et al., 2011; Gottlieb et al., 2012; Xu and Wang, 2013; Cai et al., 2015; Herrero-Zazo et al., 2015; Guo et al., 2016; Noor et al., 2016; Abdelaziz et al., 2017; Personeni et al., 2017; Piñero et al., 2017) *Validation* (Gurulingappa et al., 2012)
	Drug information databases	*Signal detection* (Tari et al., 2010; Huang et al., 2011; Boyce et al., 2014; Cheng and Zhao, 2014; Iyer et al., 2014; Cai et al., 2015; Herrero-Zazo et al., 2015; Koutkias and Jaulent, 2015, 2016; Noor et al., 2016; Zhang et al., 2016; Abdelaziz et al., 2017; Knowledge Base Workgroup of the Observational Health Data Sciences and Informatics (OHDSI) Collaborative, 2017) *MoA identification/analysis* (Lin et al., 2010; Tari et al., 2010; Huang et al., 2011; Gottlieb et al., 2012; Cai et al., 2015; Herrero-Zazo et al., 2015; Noor et al., 2016; Zhang et al., 2016; Abdelaziz et al., 2017)
	Bibliographic databases	*Signal detection* (Tari et al., 2010; Gurulingappa et al., 2012; Boyce et al., 2014; Shang et al., 2014; Zhang et al., 2014, 2016; Bravo et al., 2016; Koutkias and Jaulent, 2016; Lowe et al., 2016; Noor et al., 2016; Knowledge Base Workgroup of the Observational Health Data Sciences and Informatics (OHDSI) Collaborative, 2017; Voss et al., 2017) *MoA identification/analysis* (Tari et al., 2010; Hur et al., 2012; Xu and Wang, 2013; Zhang et al., 2016; Cañada et al., 2017; Piñero et al., 2017)
	Clinical trials data	*Signal detection* (Huang et al., 2011; Boyce et al., 2014; Koutkias and Jaulent, 2015; Knowledge Base Workgroup of the Observational Health Data Sciences and Informatics (OHDSI) Collaborative, 2017) *MoA identification/analysis* (Huang et al., 2011)
Emerging data sources	EHRs	*Signal detection* (Ceusters et al., 2011; Boyce et al., 2014; Zhang et al., 2014; Declerck et al., 2015; Jiang et al., 2015; Henriksson et al., 2016; Noor et al., 2016; Yuksel et al., 2016; Knowledge Base Workgroup of the Observational Health Data Sciences and Informatics (OHDSI) Collaborative, 2017; Personeni et al., 2017; Voss et al., 2017) *CDSS development* (Gottlieb et al., 2012; Koutkias et al., 2012; Neubert et al., 2013; Doulaverakis et al., 2014)
	Clinical narratives	*Signal detection* (Iyer et al., 2014; Zhang et al., 2014; Henriksson et al., 2015, 2016; Sarker and Gonzalez, 2015; Iqbal et al., 2017)
	Biochemical and genetic information databases	*Signal detection* (Arikuma et al., 2008; Tari et al., 2010; Boyce et al., 2014; Cai et al., 2015; Kawazoe et al., 2016; Noor et al., 2016; Abdelaziz et al., 2017; Knowledge Base Workgroup of the Observational Health Data Sciences and Informatics (OHDSI) Collaborative, 2017) *MoA identification/analysis* (Arikuma et al., 2008; Tari et al., 2010; Gottlieb et al., 2012; Hur et al., 2012; Cai et al., 2015; Noor et al., 2016; Abdelaziz et al., 2017; Piñero et al., 2017)
	SPLs	*Comparative drug analysis* (Bisgin et al., 2011; Boyce et al., 2013, 2014) *MoA identification/analysis* (Gottlieb et al., 2012; Cai et al., 2015; Guo et al., 2016; Abdelaziz et al., 2017) *CDSS development* (Neubert et al., 2013; Doulaverakis et al., 2014)
	Social media	*ADE information collection* (Liu and Chen, 2015; Nikfarjam et al., 2015; Sarker and Gonzalez, 2015; Eshleman and Singh, 2016; Liu et al., 2016, 2018; Audeh et al., 2017; Cocos et al., 2017; Nguyen et al., 2017)

*Established data sources* SRSs constitute the dominant data source for DS. They have been widely used in the reviewed articles for signal identification (mostly through NLP) as well as monitoring and validation. Interestingly, in order to improve the mining capacity of FAERS for signal detection and promote semantic interoperability between FAERS and other data sources, NLP techniques and normalization procedures were applied to FAERS data using reference terminologies, i.e., MedDRA, RxNorm, and the National Drug File—Reference Terminology (NDF-RT) (Wang et al., 2014).

ADE databases [mostly SIDER, the Comparative Toxicogenomics Database (CTD), and MetaADEDB], clinical trials data (from ClinicalTrials.gov), drug information databases (e.g., DrugBank) and bibliographic databases [i.e., PubMed/MEDLINE and the Semantic MEDLINE Database (SemMedDB) (Kilicoglu et al., 2012), a database of semantic relationships extracted from MEDLINE] have been employed for signal detection and MoA investigation.

*Emerging data sources* Observational healthcare databases and Electronic Health Records (EHRs) in particular, gained a major interest recently for DS research. In the scope of KE for DS, structured EHRs were used for signal detection, combining the use of ontologies and NLP approaches, as well as for developing medication-related Clinical Decision Support Systems (CDSSs). Unstructured EHR data, i.e., free-text clinical notes, were also used for ADR identification.

Recent advances in high-throughput sequencing technologies enable the integration of biological information to support SP by focusing on gene-drug-disease interaction networks. An increasing number of these frameworks incorporate genetic data (most often genomic polymorphisms as described in PharmGKB) for drug-drug interactions (DDIs) and ADR *in silico* prediction, stressing the need to integrate such data to complement *in vivo* and *in vitro* investigations on pharmacogenomics. Information on pathways (e.g., from KEGG), proteins (e.g., from UniProt) and their annotations with GO were the most prominent data sources for ADE identification and the analysis of the respective MoA. Interestingly, the use of biomolecular functional network data improved ADR predictions (Huang et al., 2011), and suggests that such prediction could help to design new models for investigating ADRs and their MoA, to avoid tedious and costly clinical trials, in line with the paradigms of *in silico clinical trials* and SP.

Structured Product Labels (SPLs) have been also used in various studies, including comparative drug analysis and the analysis of drug MoA. Furthermore, national SPL indexes were used as a data source for localized CDSSs.

Social media platforms (mostly Twitter, DailyStrength.com, and dedicated patient forums) attracted recently major interest for DS. Exploiting KE activities like knowledge extraction in social media can add a valuable new data source in the DS ecosystem, as they are characterized by three interesting aspects (Koutkias et al., 2017): (a) they provide vast amounts of data, (b) posts could be monitored across time and trends could be identified in relation with triggering events (e.g., new safety issues reported by regulatory authorities or announced in the media), and (c) user interconnections (e.g., mentions, responses, followership, etc.) could create a “social graph” which could provide useful insights through graph-based Social Network Analysis (SNA). Notably, a comparative study concerning the prevalence of ADR mentions in Twitter and other social media platforms concluded that social media can be considered as a valuable data source for DS (Nguyen et al., 2017).

##### Knowledge sources

Table 3 summarizes the use of the most prominent knowledge sources in the reviewed articles, citing indicative references^21^. We categorize them into reference terminologies, thesauri, and vocabularies, spanning from simple hierarchies to ontologies, which express richer semantics.

**Table 3:** Use of the most prominent knowledge sources in the reviewed articles.

	**Knowledge source**	**Use in the reviewed articles**
Terminologies/Thesauri/Vocabularies	MedDRA/WHO-ART	*Semantic annotation of concepts (grouping, classification etc.)* (Henegar et al., 2006; Neubert et al., 2013; Courtot et al., 2014; Declerck et al., 2015; Jiang et al., 2015; Guo et al., 2016; Xie et al., 2016a,b; Cai et al., 2017; Segura-Bedmar and Martínez, 2017; Voss et al., 2017) *Reference terminology for data integration* (Sarntivijai et al., 2012, 2016; Boyce et al., 2014; Yuksel et al., 2016; Knowledge Base Workgroup of the Observational Health Data Sciences and Informatics (OHDSI) Collaborative, 2017) *NLP (e.g., Named Entity Recognition)* (Bisgin et al., 2011; Gurulingappa et al., 2012; Iyer et al., 2014; Cai et al., 2015)
	UMLS	*NLP (e.g., Named Entity Recognition)* (Segura-Bedmar et al., 2010, 2011; He et al., 2013; Kang et al., 2014; Shang et al., 2014; Jiang et al., 2015; Sarker and Gonzalez, 2015; Eshleman and Singh, 2016; Liu et al., 2016, 2018; Zhang et al., 2016) *Reference terminology for data integration* (Henegar et al., 2006; He et al., 2013; Boyce et al., 2014; Cheng and Zhao, 2014; Iyer et al., 2014; Cai et al., 2015; Bravo et al., 2016; Noor et al., 2016; Abdelaziz et al., 2017; Cohen and Widdows, 2017; Piñero et al., 2017)
	ATC	*Reference terminology for data integration* (Gottlieb et al., 2012; Koutkias et al., 2012; Cheng et al., 2013; Neubert et al., 2013; Kawazoe et al., 2016) *NLP (e.g., Named Entity Recognition)* (Bisgin et al., 2011; Henriksson et al., 2016; Segura-Bedmar and Martínez, 2017) *Semantic annotation of concepts (grouping, classification etc.)* (Lin et al., 2010; Cheng and Zhao, 2014; Doulaverakis et al., 2014; Iyer et al., 2014; Cai et al., 2015; Abdelaziz et al., 2017; Personeni et al., 2017)
	RxNorm	*Reference terminology for data integration* (Boyce et al., 2014; Iyer et al., 2014; Wang et al., 2014; Jiang et al., 2015; Cai et al., 2017; Hogan et al., 2017; Knowledge Base Workgroup of the Observational Health Data Sciences and Informatics (OHDSI) Collaborative, 2017; Personeni et al., 2017; Voss et al., 2017)
	ICD-9/10	*Reference terminology for data integration* (Koutkias et al., 2012; Boyce et al., 2014; Declerck et al., 2015; Yuksel et al., 2016) *Semantic annotation of concepts (grouping, classification etc.)* (Huang et al., 2011; Zhang et al., 2013; Doulaverakis et al., 2014; Henriksson et al., 2015; Personeni et al., 2017)
	SNOMED-CT	*Semantic annotation of concepts (grouping, classification etc.)* (Iyer et al., 2014; Henriksson et al., 2015; Guo et al., 2016; Personeni et al., 2017) *Reference terminology for data integration* (Zhang et al., 2013; Boyce et al., 2014; Declerck et al., 2015; Yuksel et al., 2016)
	MeSH	*NLP (e.g., Named Entity Recognition)* (Kang et al., 2014; Henriksson et al., 2015; Lowe et al., 2016; Knowledge Base Workgroup of the Observational Health Data Sciences and Informatics (OHDSI) Collaborative, 2017; Piñero et al., 2017; Voss et al., 2017) or manually (Cheng and Zhao, 2014; Bravo et al., 2016)
Ontologies	OAE/VAE	*Combined with disproportionality analysis for signal detection and comparative drug analysis* (Sarntivijai et al., 2012; Xie et al., 2016a,b; Wang et al., 2017) *Basis for other ontologies* (Tao et al., 2012; Marcos et al., 2013; Lin and He, 2014; Herrero-Zazo et al., 2015; Guo et al., 2016; Liu et al., 2017; Wang et al., 2017) *Enhance NLP results* (Gurulingappa et al., 2012; Hur et al., 2012)
	OntoADR	*Combined with OAE to investigate MoA of Tyrosine Kinase Inhibitors* (Sarntivijai et al., 2016) *Secondary use of EHRs and observational studies data (e.g., signal detection and automatic report generation)* (Declerck et al., 2015; Yuksel et al., 2016) *Searching, coding, and information retrieval of ADE information* (Bousquet et al., 2014; Souvignet et al., 2016)

*Reference terminologies, thesauri, and vocabularies* Several knowledge sources [e.g., UMLS, MedDRA, ATC, RxNorm, ICD, SNOMED-CT, and Medical Subject Headings (MeSH)] were used as reference terminologies for knowledge extraction through Named Entity Recognition (NER), which is a typical step in NLP applications. Furthermore, they provided a “light” semantic structure of concepts (i.e., a concept hierarchy), which could be exploited for automatic inference. One of their most prominent uses was the semantic normalization of heterogeneous data sources during data integration. For example, UMLS was widely used in knowledge extraction activities, i.e., as reference terminology in NER steps applied to recognize entities in free-text through the MetaMap-MMtx tool, to reduce the semantic ambiguity between the various data sources. MedDRA and the World Health Organization (WHO) Adverse Reaction Terminology (WHO-ART) were used to semantically categorize and interrelate (e.g., group) concepts regarding signals or ADE reports and also as common reference terminologies for integration purposes or NLP tasks. In US-originated studies, RxNorm was used as a reference terminology for drugs, but to a smaller extent compared to ATC overall. An interesting application of SNOMED-CT was for enhancing the semantics provided by WHO-ART (Alecu et al., 2008) and MedDRA (Bousquet et al., 2014; Dupuch and Grabar, 2015).

*Ontologies* OAE (He et al., 2014b) and VO (Lin and He, 2012; Zhang et al., 2013) constitute reference ontologies in the domain. They were combined with statistical approaches and disproportionality analysis for the comparative analysis of drugs and ADE profiles. OAE and VO were also used to enhance the results of plain NLP algorithms, or as a conceptual base for other ontologies like the Ontology of Vaccine Adverse Events (OVAE), the Ontology of Drug Neuropathy Adverse Events (ODNAE), the Ontology of Cardiovascular Drug AEs (OCVDAE), the Ontology of Chinese Medicine for Rheumatism (OCMR), and the Ontology of Genetic Susceptibility Factors (OGSF). Furthermore, OAE has been identified as an ontology which could support a systems-based modeling approach for regulatory drug approval purposes (Zhichkin et al., 2012; Sinha et al., 2016).

The RxNorm-based Drug Ontology (DrOn) represents the therapeutic functions of drug products, including their MoA at the molecular level and their adverse effects (Hogan et al., 2017). However, it seems that it is not extensively employed for DS purposes, as DS was not among its main use cases.

Notably, OntoADR is an ontologized version of MedDRA (Bousquet et al., 2014), which was used in the SALUS project to integrate MedDRA in an overall ontology-based information model and support secondary use of EHR data for DS (Declerck et al., 2015) and observational studies (Yuksel et al., 2016). Similarly, OAE and MedDRA have been interlinked to investigate the biological mechanisms of Tyrosine Kinase Inhibitors (Sarntivijai et al., 2016).

#### Knowledge Engineering Activities

In this subsection, we present how the main KE activities were employed in the reviewed articles and highlight the most prominent approaches. Thus, we emphasize on the employed KE methods, illustrating how these were employed for DS.

##### Knowledge dissemination

A platform aiming to facilitate knowledge dissemination regarding drug safety, efficacy, and effectiveness was proposed, overcoming the issue of outdated drug product labels (Boyce et al., 2013). The study integrated many data sources in a single knowledge graph containing information related with drug products (including ADEs and DDIs) and provided a proof-of-concept Web interface allowing to actively explore all the information related with a specific drug product. Knowledge dissemination approaches were also employed to support comparative drug analyses regarding ADEs and contraindications, using visual analytics combined with ontological reasoning (Lamy et al., 2017).

##### Knowledge elicitation

Knowledge elicitation activities are typically related with rule-based inferencing combined with ontological reasoning methods. For instance, a conceptual model relying on the Drug Interaction Ontology (DIO) to identify DDIs was developed based on two rule-based inferencing modules (Pathway object constructor and Drug interaction detector) (Arikuma et al., 2008). Drug-Drug Interactions Ontology (DINTO) combined Description Logic (DL) (Baader et al., 2004) based reasoning with rules formed in the Semantic Web Rules Language (SWRL) to identify DDIs and investigate their MoA (Herrero-Zazo et al., 2015), upon a conceptual model exploiting Pharmacokinetics and Pharmacodynamics related knowledge. The Drug Enzyme Interaction (DEI) ontology was combined with a rule-base to investigate drug MoAs (Zhang et al., 2016). Similarly, ProLog was used to encode rules regarding drug metabolism and conduct reasoning to identify potential DDIs (Tari et al., 2010). In addition, SPARQL queries following specific patterns regarding temporal inference were used to identify ADRs upon HL7 messages integrated in one large Resource Description Framework (RDF) graph (Kawazoe et al., 2016). Rules referring to four levels of interaction mechanisms, namely, pharmacokinetic, pharmacodynamic, pharmacogenetic, and multi-pathway interaction, were employed to identify DDIs and their underlying MoAs upon a large RDF knowledge graph integrating 15 DDI databases (Noor et al., 2016).

Inferencing methods based on graph theory were also extensively applied. Graph clustering coefficient analysis was used to identify similar ADE clusters (Lin et al., 2010). Node closeness in a protein–protein interaction graph was used to infer DDIs (Gottlieb et al., 2012), while network centrality was investigated in a gene-gene interaction graph as a metric of gene importance in terms of causing fever (Hur et al., 2012). Several graph-based metrics (i.e., connectivity, betweenness, and clustering coefficient) were used to predict ADEs in a knowledge graph built upon MetaADEDB (Cheng et al., 2013). Graph shortest paths were used to identify the weight of relationships in a vaccine-related network extracted from SemMedDB, to confirm the structural validity of VO (Zhang et al., 2013). A similar approach was used to identify relationships between drugs and ADE terms presented in the UMLS Metathesaurus semantic network, in order to extract ADEs from biomedical text (Kang et al., 2014). A graph kernel based ML approach was used to extract drug-enzyme relationships from the literature, using UMLS as reference terminology (Zhang et al., 2016). Graph-based metrics combined with terminological reasoning were employed to calculate the semantic distance between MedDRA terms and cluster them to improve Standardized MedDRA Queries (SMQs) (Dupuch and Grabar, 2015). The relationships of drugs and their effects were modeled in the form of the so-called Drug Effect Graph and used topological characteristics to identify ADE relations in Twitter (Eshleman and Singh, 2016).

DL-based reasoning upon ontologies was applied in various cases (Vandervalk et al., 2013; Zhang et al., 2013; Courtot et al., 2014; Herrero-Zazo et al., 2015; Souvignet et al., 2016; Lamy et al., 2017). In particular, combining the use of ontology reasoning (upon OAE and VO) with more traditional disproportionality measures like the Proportional Reporting Ratio (PRR) was used to analyse already identified ADEs and interrelate the statistic properties of each signal with the categorical information provided by the respective ontologies (Sarntivijai et al., 2012; Xie et al., 2016b; Wang et al., 2017). A similar approach, combining ontology reasoning upon OAE interlinked with MedDRA and disproportionality analysis of SRS data (i.e., FAERS and VAERS) was presented in Sarntivijai et al. (2016) and Xie et al. (2016a).

Terminological reasoning was combined with ontologies and other statistical approaches, including disproportionality analysis. For example, an advanced association rule mining approach was presented for identifying causality between drugs and ADEs in FAERS (Cai et al., 2017). In particular, the Relative Reporting Ratio (RRR) was used to model confidence as defined in association rule mining, combined with terminological reasoning based on RxNorm and MedDRA upon FAERS data.

ML was also identified as a prominent paradigm employed for knowledge elicitation. SVMs were used in several classification schemes (Huang et al., 2011; Henriksson et al., 2016; Zhang et al., 2016), while association rules were elicited and contextualized in Koutkias et al. (2012) for ADE prevention based on EHR data. Vector-based similarity mechanisms were also extensively used, mostly for content-based document classification (Henriksson et al., 2015; Nikfarjam et al., 2015; Cocos et al., 2017). For example, SemMedDB predicates (i.e., triplets in the form of subject-predicate-object) were modeled as vectors and used an SVM to classify concepts (Cohen and Widdows, 2017), while deep-learning neural networks were used to identify ADEs in Twitter (Cocos et al., 2017). A vector-based approach implemented pattern structures, in combination with the class hierarchies of three medical ontologies (ICD-9-CM, SNOMED-CT, and ATC), to mine association rules that characterize ADEs occurring in distinct patient subgroups (Personeni et al., 2017).

Finally, a large-scale DDI prediction system relying on a large RDF knowledge base was developed upon vector-based as well as graph-based similarity metrics combined with terminological reasoning (Abdelaziz et al., 2017).

##### Knowledge extraction

The most widely used knowledge extraction approach refers to the use of NLP techniques applied on unstructured data, i.e., free-text, originated from biomedical literature, social media, clinical notes, etc., using various computational methods (including ML-based).

Relying on core NLP methods, the DrugNerAR system demonstrated its ability to identify drug mentions in biomedical literature for DDI identification (Segura-Bedmar et al., 2010), and drug-gene relationships, extracted from MEDLINE (Xu and Wang, 2013). NLP was also used upon bibliographic data sources, storing a structured representation of plain text in a “parse tree database” for further elaboration and reasoning to identify DDIs (Tari et al., 2010). Notably, an alternative approach targeting social media took into account the three previous and the three next tokens to analyse each token in its context for identifying ADR mentions (Nikfarjam et al., 2015). Context-based semantic analysis across sentences improved the identification of ADRs in patient forums, using the NegEx tool and drug indications to filter out negated ADEs and drug indications, respectively (Liu and Chen, 2015). NLP was also applied on clinical notes to identify DDIs based on drug-gene relationships extracted from SemMedDB (Zhang et al., 2014), while SemMedDB was also exploited for Literature Based Discovery aiming at signal assessment (Shang et al., 2014). Similarly, NLP was applied on the clinical notes of a large dataset, taking into account contextual information (i.e., temporal information and categorization in factual, hypothetical or negated sentence), to detect ADEs specific to antipsychotics and antidepressants (Iqbal et al., 2017). NLP was also applied on WikiPedia to identify drugs and conditions in the title of its articles, as well as links to other pages related to drugs, conditions and ADRs, aiming to construct a lexicon of ADR terms (Lowe et al., 2016).

An alternative approach used topic modeling on free-text drug leaflets to generate novel hypotheses regarding DS (Bisgin et al., 2011). Topic modeling and sentiment polarity were used as contextual information regarding the identification of ADEs in Twitter (Eshleman and Singh, 2016). Ontology-assisted NLP was used to identify ADE mentions in free-text sources, i.e., medical case reports and literature, targeting at signal identification (Gurulingappa et al., 2012). Finally, SPLs were used to extract information and integrate it in a large RDF graph (Boyce et al., 2013).

On exploiting ML-based approaches, the SSEL-ADE framework relied on an SVM employing n-grams and graph-based metrics to identify ADE mentions in social media (Liu et al., 2018). N-gram models were used combining 3 SVM kernels and stacked generalization to improve the identification of DDIs in biomedical literature (He et al., 2013). An ensemble of ML methods was employed to identify DDIs in clinical narratives, taking into account contextual information for the analysis of each term (i.e., negation, speculation and temporality) (Henriksson et al., 2015, 2016). Notably, third-party data sources were integrated in one knowledge base combined with ML to identify ADEs in biomedical literature (Bravo et al., 2016). Interestingly, crowdsourcing was used to manually annotate a corpus of free-texts (in a reasonable time and without bias) to train the ML model.

##### Knowledge integration

WHO-ART and SNOMED-CT were mapped based on synonymy in the UMLS Metathesaurus to automatically generate definitions of WHO-ART terms in a DL formalism, i.e., the Web Ontology Language (OWL), aiming to identify WHO-ART terms that may be grouped together (Alecu et al., 2008). As the same medical condition may be coded with different terms in DS databases, it was assumed that such approach would enable to group similar terms and improve signal generation. As a next step in the same line of work, SNOMED-CT was used to convert MedDRA to an OWL ontology, namely, OntoADR, which combined the semantics of MedDRA and SNOMED-CT (Bousquet et al., 2014), through a relational database implementation (Souvignet et al., 2016).

Koutkias and Jaulent investigated the limitations of computational signal detection methods when applied on single data sources, and elaborated on multiple heterogeneous signal detection methods, data sources and other drug-related resources under a common, integrated framework (Koutkias and Jaulent, 2015). The framework relied on the Pharmacovigilance Signal Detection Ontology (PV-SDO) and a multiagent system, implementing a comprehensive workflow comprising of method selection and execution, as well as outcomes' aggregation, filtering, ranking and annotation (Koutkias and Jaulent, 2016).

Declerck et al. proposed an ontology-based abstraction layer called Common Information Model—CIM (Declerck et al., 2015). CIM was populated through software “bridges” based on mappings of local EHR databases to CIM, thus accommodating the dependencies of the overall framework on the local EHR data schemas.

Furthermore, various data sources (SPLs, ADE information, clinical trials data, etc.) were integrated in a single knowledge graph based on common-terms matching and mappings to reference terminologies, in order to provide a unified and semantically enhanced knowledge base for information regarding drug products (Boyce et al., 2013).

Considering the integration of biochemical data for DS, several sources, such as UMLS, DrugBank, CTD, and UniProt were integrated in one large RDF graph for ADR detection (Abdelaziz et al., 2017). Several heterogeneous data sources were also integrated to interrelate biochemical and phenotypic information for predicting ADEs through an SP approach (Huang et al., 2011; Cheng and Zhao, 2014). Furthermore, the Adverse Drug Reaction Classification System (ADReCS) combines a hierarchical structure of concepts (similar to the MedDRA structure) and integrates information from a large number of ADE and biochemical data sources, explorable through a Web interface for signal assessment (Cai et al., 2015). Similarly, DisGeNet is a comprehensive centralized repository created by integrating data from curated databases and two datasets obtained by mining the scientific literature (Piñero et al., 2017). It focuses on the associations between genes/variants and diseases. DS is one of DisGeNet's main use cases and can also be considered as a large knowledge graph as it is also available in RDF format (Queralt-Rosinach et al., 2016).

Regarding medication-based CDSSs, Koutkias et al. integrated various knowledge sources using the Computerized Interpretable Guideline (CIG) formalism (Koutkias et al., 2012); they used meta-rules to integrate these sources and well-defined communication interfaces, in order to satisfy both performance requirements and also the need to obtain knowledge from third-party sources. In the same context, a combination of rule-based and ontology-based knowledge representation was developed to accommodate the need for integrating various data sources and also providing effective CDSS support to prevent ADEs in a computationally effective manner (Doulaverakis et al., 2014).

The D3 (Drug-drug interactions Discovery and Demystification) system aimed to infer MoAs for DDIs based on an integrated RDF schema of 12 biomedical resources and 15 DDI databases (Noor et al., 2016). Some data sources included data in RDF format obtained from Bio2RDF, which were semantically aligned through the use of UMLS and a set of specific relationships (e.g., “has indication”). Non-UMLS compatible data sources were also integrated via explicit database cross-references.

The LAERTES knowledge base which was built in the context of the Observational Health Data Sciences and Informatics (OHDSI) collaborative (Knowledge Base Workgroup of the Observational Health Data Sciences and Informatics (OHDSI) Collaborative, 2017), integrated multiple data sources into a common knowledge schema for signal investigation, in compliance with the OMOP Common Data Model (CDM) (Boyce et al., 2014; Voss et al., 2017).

##### Knowledge representation

Ontologies are the most commonly used knowledge representation formalism and, therefore, several ontologies were introduced targeting the domain of DS, mostly using OWL and RDF.

As regards the ADE representation, OAE is the most prominent ontology. OAE is a community-based outcome, widely used to semantically categorize ADEs (He et al., 2014b). Respectively, VO is a community-based ontology used to semantically categorize vaccines (Hur et al., 2012; Lin and He, 2012; Zhang et al., 2013), typically used in combination with OAE. VO was also used in combination with the Time Event Ontology (TEO) which was developed to formally represent the time-oriented aspects of an ADE report (Tao et al., 2012), as time has been recognized as an important aspect of ADEs (Iqbal et al., 2017; Personeni et al., 2017). VO and OAE were also used as the conceptual base of OVAE to depict relationships between vaccines, adverse events, and patient age groups (Marcos et al., 2013), in the context of the VIOLIN vaccine safety analysis system (He et al., 2014a), and to classify and update data regarding ADEs of Hepatitis vaccines (Xie and He, 2017). VO and OAE were referenced by OGSF, aiming to model the genetic susceptibility (or predisposition) to vaccine adverse events (Lin and He, 2014). Furthermore, ODNAE extends OAE to facilitate the analysis of drugs causing neuropathy adverse events (Guo et al., 2016). Similarly, OCVDAE extends OAE to facilitate the analysis of ADEs caused by cardiovascular drugs (Wang et al., 2017), and OCMR extends OAE to facilitate the comparative analysis of traditional Chinese drugs regarding rheumatism (Liu et al., 2017).

Henegar et al. modeled MedDRA using DAML + OIL (OWL's predecessor) to support automatic signal generation (Henegar et al., 2006). The same group created an OWL ontology to enrich the formal definitions of WHO-ART terms with associative relations provided by SNOMED-CT to support grouping of WHO-ART terms related to the same medical condition (Alecu et al., 2008) and, as a further step, presented an ontologized version of MedDRA, exploiting SNOMED-CT semantics (Bousquet et al., 2014). OWL was also used to model ADEs based on concepts concerning the patient's medical history and their time-related aspects (Ceusters et al., 2011). Moreover, the Adverse Event Reporting Ontology (AERO) was proposed to enable the modeling of case definitions related to adverse events following immunization to support the respective information processing workflow (Courtot et al., 2014).

In the scope of representing drug interactions, DIO models drug metabolic pathway related concepts, including information from organ to molecular level, supporting SP approaches (Arikuma et al., 2008). DEI models the interactions of drugs and enzymes, used to infer potential DDIs from biomedical literature (Zhang et al., 2016). DINTO provides a DDI classification schema and a conceptual model taking into account both the pharmacokinetic and pharmacodynamic aspects of DDIs (Herrero-Zazo et al., 2015). DINTO references OAE and integrates knowledge from other data sources (i.e., ChEBI, DrugBank, and SIDER) with no manual curation, following the NeOn KE methodology (Suárez-Figueroa et al., 2012).

In a few cases, the RDF representation formalism was used without aiming to formulate a specific conceptual model; for example, HL7 messages were converted to RDF and integrated to a large RDF model to confirm that they could be used in the context of ADR detection (Kawazoe et al., 2016).

Alternatively, relational databases were used as a knowledge base storage formalism, since they provide a mature data storage paradigm, able to support vast data storage in a computationally effective manner that is widely used in real-world enterprise systems. Compared to ontologies, relational databases are not specifically designed to support KE activities (e.g., automatic reasoning). On the other hand, while ontologies can support formal semantics and automatic reasoning given their underlying robust mathematical background, i.e., DL, the respective data storage systems are not yet mature enough and the automatic reasoning process is computationally expensive for large knowledge graphs, making relational databases a competing alternative for large knowledge bases. To this end, MEDLINE abstracts were used to extract knowledge on drug metabolism and interactions (storing the corresponding structured representation into a database in the form of a tree-structure representation) and queried to identify DDI mentions (Tari et al., 2010). SemMedDB contains statements in the form of triples (subject-predicate-object) extracted from MEDLINE and stored in a relational format (Zhang et al., 2014). MetaADEDB relies on a relational schema to integrate several heterogeneous data sources for DS (Cheng et al., 2013).

Hybrid data storage approaches have been also proposed, using both relational and RDF formalisms. For example, LAERTES used relational databases as its basic data storage paradigm (Boyce et al., 2014; Knowledge Base Workgroup of the Observational Health Data Sciences and Informatics (OHDSI) Collaborative, 2017). However, it also employs the Web Annotation Data Model (WADM), to enable “drill-down” into evidence supporting a statistic measure of association between a drug and a Health Outcome of Interest (HOI) (e.g., a count, PRR, etc.). DisGeNet is also available both in relational and RDF version, accompanied by an ontology which defines its conceptual model.

#### Impact of Knowledge Engineering on Drug Safety

In this subsection, we highlight the contribution that the employed KE approaches have in DS core activities (Table 1). In particular, the emphasis is given on illustrating the value of adopting KE approaches for DS and their potential application in current DS practice.

##### ADE information collection

Currently, information collection methods to support routine DS activities (e.g., signal identification) are mostly focusing on SRS, bibliographic, and clinical trial data. In particular, bibliographic search is typically conducted manually by experts, requiring the formulation of the respective query (i.e., define the terms of interest, expand the query through synonyms, etc.), and the manual evaluation of the returned results based on expert tacit knowledge. On the other hand, via the formalization of knowledge in an explicit way, the use of KE tools can automate this process, facilitate the exploitation of new/emerging data sources, and reduce errors in the process.

Bibliographic data sources were used to extract DDIs (Segura-Bedmar et al., 2010; Tari et al., 2010; He et al., 2013) and ADE mentions (Gurulingappa et al., 2012; Kang et al., 2014). NLP combined with disproportionality analysis was used to identify DDIs in free-text clinical notes, concluding that the narrative part of EHRs can complement existing sources for post-marketing DDI surveillance (Iyer et al., 2014). Similarly, clinical narratives were exploited for ADE identification (Zhang et al., 2014; Henriksson et al., 2015). Notably, psychiatric clinical notes were used to identify ADEs achieving an F-score of 0.83 (Iqbal et al., 2017). EHR data were also used to generate ADE reports automatically, aiming to address ADE underreporting by clinicians (Declerck et al., 2015).

Various studies exploited social media with promising results^22^. In particular, they were used to identify ADE mentions using various NLP techniques (Nikfarjam et al., 2015; Sarker and Gonzalez, 2015), concluding that since the language used is highly informal, the use of context and sentiment analysis could further improve the results. A combination of statistical learning and semantic filtering improved the recognition of known ADRs in patient forums with precision ranging between 75 and 82% and recall between 56.5 and 65.3% (Liu and Chen, 2015). High accuracy in recognizing ADE mentions in two MedHealth forums and Twitter were also reported, with area under the curve (AUC) values of 84.5, 77.3, and 84.5%, respectively (Liu et al., 2016). Finally, a graph-based inference approach combined with topic modeling and sentiment analysis identified adverse drug effect mentions in Twitter with precision exceeding 85% and F1 exceeding 81% (Eshleman and Singh, 2016).

##### ADE detection

Systematic approaches for knowledge extraction, integration and further processing (e.g., based on DL reasoning) demonstrated promising results on ADE detection. An exemplar implementation of *in silico* DDI prediction incorporating drug metabolic pathways and molecular events enabled the quantitative evaluation of drug interactions (Arikuma et al., 2008). A prototype implementation was able to quantitatively examine the effect of irinotecan-ketoconazole interactions using numerical simulations. The extension of this method for other drug pairs as well as multiple drug interactions showed the potential to support computational DDI predictions using DIO. As a result, four potential drug interactions that involved cytochrome p450 (oxidation by CYP3A4) and drug binding reaction to albumin were automatically detected via DIO, while two of them had not been reported in the literature. DDIs were successfully identified (>75% according to the presented evaluation scheme) by modeling the behavior of regulatory elements, particularly enzymes (Tari et al., 2010). Furthermore, live attenuated influenza vaccines were found to have lower chance of inducing Guillain-Barre Syndrome and paralysis than trivalent (killed) inactivated influenza vaccine (Sarntivijai et al., 2012).

Integrated knowledge bases created with the support of KE processes demonstrate remarkable results regarding ADE detection. The ability to identify ADEs through large-scale data integration in one knowledge base was demonstrated using MetaADEDB (Cheng et al., 2013). Using FAERS as the gold standard during the evaluation process, MetaADEDB facilitated ADE detection (AUC value reported more than 0.9 by 10-fold cross validation and 0.912 for external validation). Furthermore, the LAERTES knowledge base (Knowledge Base Workgroup of the Observational Health Data Sciences and Informatics (OHDSI) Collaborative, 2017) was evaluated including positive and negative controls, illustrating an AUC value of 0.92 (Voss et al., 2017).

Notably, INferring Drug Interactions (INDI) inferred both pharmacokinetic and pharmacodynamic DDIs upon EHR data by applying ML on drug MoA similarity and their biochemical properties (Gottlieb et al., 2012). Its validation confirmed one of the predicted CYP-related DDIs using hospital data in Israel. Finally, Tiresias, a DDI prediction system relying on a large integrated RDF knowledge base, was successfully used to predict DDIs, identifying 68% of all DDIs found after 2011, using only information about DDIs present in the January 2011 version of DrugBank (Abdelaziz et al., 2017).

##### ADE assessment

ADE assessment mostly refers to the analysis of the underlying MoA as well as the comparative analysis of drugs. These activities typically require the integration of heterogeneous data sources, including biochemical and genetic information databases.

Dynamic reconstruction of drug metabolic pathways from primitive molecular events using information modeled in DIO was conducted, showing that unknown potential pathways can be inferred through the combination of ontologies and rule-based inference (Arikuma et al., 2008). Similarly, drug target information was used to identify clusters of similar DDI cases reported in FAERS and provide explanations for their MoA (Lin et al., 2010). The ability to interpret the MoA of the respective DDIs was demonstrated by exploiting drug metabolism knowledge encoded in the form of rules linking proteins and drugs via four types of relationships (i.e., metabolizes, induces, inhibits, regulates) (Tari et al., 2010). For each DDI identified, the respective triggered rules could be considered as a description of the respective MoA. Alternatively, a gene interaction graph regarding vaccines was built based on bibliographic data, and provided a method to identify genes potentially related with the ADE of fever (Hur et al., 2012). In DIO, drug-enzyme relationships were used to model the mechanism of drug metabolism for DDI detection in biomedical literature, achieving an F-measure of 84.97% for drug-enzyme relationships recognition and 83.19% for DDI recognition against the “*in vivo*” dataset used for evaluation (Zhang et al., 2016). Finally, in the context of the eTOX project a Web application was presented, aiming to facilitate the exploration of a knowledge base regarding drugs, genes and compounds' toxicity associations for investigating liver toxicity (Cañada et al., 2017).

An interesting contribution was the development of a semantics-enabled Web analytics tool, namely, the Case Series Characterization Tool (CSCT) (Yuksel et al., 2016). CSCT has been used to conduct observational studies and comparative drug analyses, exploiting the integration of semantically and syntactically heterogeneous data sources, addressed by an ontology-based data information model. The CSCT deployment was validated by PV researchers from both UMC and the Lombardy Regional Pharmacovigilance Centre. The main advantages of the presented approach are: (a) easier definition of analysis rules (since CIM semantics were independent of the underlying data sources' syntactic or semantic schema), and (b) scalability of the proposed integration model due to semantic mediation of CIM as “*whenever a new source or target content model is to be added, the required mapping to the CIM is added in linear time, without affecting the existing resources*.”

Another notable contribution of the reviewed studies concerns the semantic enhancement of widely used terminologies like MedDRA. OntoADR (semantically) enhanced MedDRA using knowledge from sources, such as SNOMED-CT (Bousquet et al., 2014; Souvignet et al., 2016). The “ontologization” of MedDRA could significantly benefit disproportionality analysis, data mining or other techniques used for post-marketing DS surveillance, since MedDRA taxonomic limitations can decrease the sensitivity and specificity of signals computed by automatic approaches (Yokotsuka et al., 2000; Bousquet et al., 2005a,b).

Furthermore, the use of ontologies and the reasoning capabilities that they offer facilitated ADE profiling. In particular, the semantics provided by OAE and VO or their extensions combined with statistical approaches (i.e., disproportionality analysis) against various DS data sources (i.e., FAERS, VAERS, drug package insert documents from the China Food and Drug Administration Website) were employed, in order to extract ontology-assisted ADE profiles and investigate the underlying MoAs (Lin and He, 2012; Guo et al., 2016; Xie et al., 2016a,b; Wang et al., 2017). Some profiles were identified as novel, since they were not previously reported in the literature [e.g., ADE profiles regarding the *M. bovis* strain Bacillus Calmette—Guerin (Xie et al., 2016a)]. Using this approach, two drug ingredient classes and three cardiovascular drug MoA classes were found to have statistically significant class effects on 13 AEs (Wang et al., 2017). The fact that valid, novel ADE profiles were automatically inferred and linked to specific MoAs through the use of ontologies, highlights the significance of adopting KE-based approaches in the context of DS.

Another significant contribution of the reviewed KE approaches regarding ADE assessment concerned the prioritization of ADE signals according to their importance. A normalized AERS dataset and the Common Terminology Criteria for Adverse Events (CTCAE) were used to prioritize DDI-induced ADEs identified in FAERS (according to their severity), as well as occurrences of medications and problems extracted from clinical notes from Mayo Clinic's EHR (Jiang et al., 2015). This ontology-based approach facilitated automatic prioritization of DDIs related to Warfarin, Clopidogrel, and Simvastatin, three frequently prescribed cardiovascular drugs.

Finally, regarding the investigation of ADE MoAs, the D3 system uses a rule-base with nine rules corresponding to nine different interaction mechanisms divided into four levels (Noor et al., 2016): pharmacokinetic (protein binding, metabolic inhibition, metabolic induction, transporter inhibition, and transporter induction); pharmacodynamic (additive-enhancement and competition); pharmacogenetic (SNPs that may alter drug exposure); and multiple pathway interactions (MPIs). For example, when both drugs x and z share at least 1 enzyme y and 1 transporter y2, then an MPI mechanism could be inferred and the rule would be “x *metabolized_by* y; x *transported_by* y2; z *metabolized_by* y; z *transported_by* y2.” The results of using such an inference mechanism included 85% recall rate and 61% precision rate in terms of the inference or lack of inference of DDI MoA explanations, for a random collection of interacting and non-interacting drug pairs, respectively.

##### ADE prevention

In the context of the ReMine project, an ontology was developed to support adverse event prevention and mitigation, in addition to detection and monitoring, based on the patient's medical history (Ceusters et al., 2011). While the ReMine project aimed to better document adverse events and facilitate the development of mitigation and prevention strategies on the long term, others were aiming at real-time interventions. For example, in the context of the PSIP (Koutkias et al., 2012), Panacea (Doulaverakis et al., 2014) and E-pharmacovigilance (Neubert et al., 2013) projects, knowledge-based DSSs were developed for preventing ADEs in the clinical environment, taking into account hospital data and also focusing on the clinical context to address aspects, such as over-alerting (Koutkias et al., 2012).

A novel Web analytics platform aimed to facilitate clinicians to conduct comparative drug analysis for ADE prevention (Lamy et al., 2017). The proposed tool was based on ontological reasoning, in order to classify information and highlight important relationships between drugs and ADEs. The tool was evaluated by 22 General Practitioners, demonstrating high rates of user acceptance.

Interestingly, few works focused on “personalized” ADE prevention. In particular, an automatic technique to identify gene-drug relationships was presented (Xu and Wang, 2013), as well as a prototype implementation of a Web browser plugin providing personalized warnings for DDIs based on ontologies and Personal Health Record (PHR) data (Vandervalk et al., 2013).

##### ADE monitoring

ADE monitoring concerns the process of tracking the evolution of an ADE through time, mostly for epidemiological reasons. As this process is mostly relevant with statistical metrics, KE approaches are not expected to significantly contribute in that and, therefore, ADE monitoring was not one of the main focuses in the reviewed papers. Notably, only one of the selected papers explicitly referred to ADE monitoring as one of its key objectives, through secondary use of EHR data (Yuksel et al., 2016).

##### ADE reporting

ADE reporting can be defined as a bidirectional activity: (a) patients and healthcare professionals (HCPs) reporting potential ADRs to regulatory agencies and the pharma industry, and (b) drug monitoring organizations or regulatory agencies communicating DS-related information (e.g., new signals or confirmed ADRs) to HCPs and patients. Both reporting channels pose challenges, e.g., under-reporting toward drug monitoring agencies, ambiguity and vast amount of information communicated to patients and HCPs, etc. These reporting processes could significantly benefit from KE approaches; however, it seems that this DS activity does not receive much attention and can be identified as a “research gap” with a lot of room for progress.

An open Web platform based on SPL information and other interlinked data sources was developed to support the dissemination of information regarding DS by exploiting comparative drug effectiveness among other information (Boyce et al., 2013). The targeted users were primarily clinicians and researchers.

An ontology-supported methodology for reporting adverse events following immunization to regulatory agencies according to the Brighton case definition was presented based on the AERO ontology (Courtot et al., 2014). The study demonstrated the feasibility of confirming automated diagnosis and concluded that a logical formalization of existing guidelines could improve reporting by identifying missing elements and enforcing consistency through standardization. The approach allows medical experts to prioritize reports and, therefore, such formalization may accelerate the identification of vaccine-induced ADRs and the response of regulatory agencies.

Interestingly, the SALUS project developed an ontology-based approach to automatically generate ADE reports from EHR data in the E2B format (Declerck et al., 2015).

### Risk of Bias

Bias is defined as a “*systematic error, or deviation from the truth, in results or inferences*” (Altman et al., 2011). Risk of bias can refer to multiple aspects of the systematic review process and can be related with various causes (Drucker et al., 2016). For example, “*evidence selection bias occurs when a systematic review does not identify all available data on a topic”* and this “*can arise from publication bias, where data from statistically significant studies are more likely to be published than those that are not statistically significant.”* It should be clarified that bias does not refer to imprecision (e.g., due to the reviewing process inherent subjectivity, further discussed in subsection Limitations), but only refers to *systematic error* introduced by the systematic review protocol.

Table 4 depicts the main bias sources and the way that our study protocol has mitigated the respective risks. It should be noted that bias risks have been investigated mostly in the context of clinical trials or similar interventions and this has also affected the widely accepted risks of bias as well as their reporting or mitigation mechanisms. As the presented review does not refer to a medical intervention, the respective bias risks and their effect on the presented study have been adapted accordingly.

**Table 4:** Analysis of bias risks and mitigation measures employed in the current study.

**Bias risk**	**Application in current study**	**Reporting/Mitigation**
*Selection bias:* missing important research because it was not published due to bias (e.g., due to lack of statistical significance)	Our systematic review focuses on qualitative criteria which cannot be statistically measured. Therefore, criteria like statistical significance could not affect our study and reporting tools like funnel plots are not applicable. However, indexing errors in the systematic review initial data source(s) could lead to missing potentially relevant articles.	We employed two reference bibliographic repositories (namely, PubMed and Web of Science), in order to mitigate the risk of missing articles due to indexing errors.
*Primary study bias:* reviewed studies could be biased regarding the evaluation mechanism used, the presented findings, conclusions, etc. (a.k.a. reporting bias)	Since there is no widely accepted methodology to publish the results of KE practices on DS application, the reviewed studies report results in an arbitrary manner that could indeed affect overall conclusions.	Identified specific evaluation and reporting weaknesses that could imply bias in the systematic review evaluation criteria. The reviewed studies that have been identified to suffer from such reporting weaknesses correspond to 62.5% of the selected articles.
*Competing interests:* reviewed studies (or even the presented systematic review) could be sponsored by companies or have other ties to industry	The authors of the presented systematic review do not have ties with industry or any other kind of relationship which could imply competing interests. Some reviewed articles originate from companies and, therefore, this kind of bias could have an implication in their reported outcomes.	The industrial participation in the studies was identified as a specific evaluation criterion. More specifically, these studies correspond to 30% of the selected articles.

## Discussion

Drug safety encompasses all data gathering and processing activities related with the detection, assessment, understanding and prevention of adverse effects throughout the entire lifecycle of drugs (World Health Organization, 2002). In a pre-market setting, clinical trials of newly developed drugs constitute the main procedure for identifying ADRs resulting from their use. However, due to time constraints, the limited population size as well as potential bias, clinical trials do not enable the detection of all possible ADRs. Consequently, post-marketing surveillance is necessary to identify new or incompletely documented ADRs throughout the time a drug is actively prescribed (World Health Organization, 2008).

Recently, several studies argued that data employed for DS should be extended from the traditional data sources, i.e., SRSs and bibliography, to observational healthcare databases and even social media platforms, while linkage with biochemical and genetic information would be desirable to provide MoA and may allow to identify more unexpected AEs. In order to achieve this advancement and take into account these requirements, DS monitoring organizations have to face new challenges, both scientific and technical, given that the above sources are not designed to serve DS aspects *per se*. In particular, there is an emerging need for high-throughput computational methods that will enable, from the one hand, efficient data analysis and interpretation and, on the other hand, knowledge extraction, representation, exploitation and management (Koutkias and Jaulent, 2015).

Up to now, the emphasis in computational DS surveillance was mostly given on data-driven and statistics-based approaches. The current review focused on KE, a discipline of Computer Science which exploits methods for acquiring, representing and exploiting knowledge, having as its cornerstone well-defined formalisms and structures. The study illustrated the methods employed and the impact that current KE-based approaches have in DS, while also highlighting trends, limitations, as well as opportunities for further research.

### Summary of Main Findings

The number of studies exploiting KE for DS increased constantly between 2006 and 2017 (Figure 3C). The reviewed articles illustrated the interest in exploiting diverse data and knowledge sources as well as the application of various KE methods, spread across the entire spectrum of the core KE activities as defined in our study (in many cases targeting multiple KE activities). Interestingly, these studies targeted diverse DS aspects as well, including both core DS activities (i.e., ADE information collection, assessment, etc.) and DS topics of special interest (e.g., vaccine safety, drug interactions, etc.), according to our study context (Table 1).

The distribution of authors across the globe (Figure 3B) illustrated an international interest in KE for DS. However, the relatively low contribution from DS monitoring organizations as well as healthcare organizations in research studies in the field (Figure 3A), could be attributed to the lack of the required KE-oriented technical expertise and, perhaps, to the reluctance in adopting technological paradigms that are not directly related with familiar approaches, e.g., statistical inference, disproportionality analysis, etc. This may also indicate a significant challenge for KE researchers in the domain to illustrate a major “success story,” which would disseminate the value of KE approaches in the context of DS, and therefore, facilitate their wider adoption. The reviewed studies illustrated mostly proof-of-concept outcomes, indicating that KE for DS is still in its infancy, especially regarding its application in routine DS activities.

While wide interest in exploiting diverse as well as emerging data sources is apparent, it raises many challenges and room for further research. For example, the biological knowledge underlying drug metabolism and pharmacological mechanisms has not been adequately elaborated to infer new causal relationships among drugs and effects. Besides polymorphic sites and alterations to gene expression, other molecular mechanisms, such as regulatory elements and epigenetic modifications, may have direct or indirect relationship with medication and consequently ADEs. Furthermore, standardization of observational healthcare data is an important issue (Koutkias, 2019). Common data models relying on reference terminologies, such as the OMOP CDM (Voss et al., 2015), may scale-up the applicability and the reproducibility of computational analysis methods in the domain. Despite the inherent noise in social media content and the complexity in analyzing it, this data source cannot be neglected due to its wide penetration in everyday life and its capacity to provide insights especially for rare health-related events (Klein et al., 2018).

To a great extent, the reviewed studies relied on publicly available data, provided, for example, via PubMed/MEDLINE and FAERS. Nevertheless, important, systematically curated and rather new Linked Data infrastructures, such as the EBI-RDF platform^23^ and OpenPHACTS^24^ are available, which were not adequately considered in the reviewed studies. In terms of knowledge sources, UMLS, MedDRA, ATC, SNOMED-CT, and ICD were the most widely used terminologies, as these constitute reference and well-curated resources with varying granularity. With respect to ontologies, OAE, VO, and GO were the most widely used, due to their rich content and relevance with DS.

In terms of KE activities, knowledge extraction and knowledge representation were extensively elaborated, while the focus on knowledge dissemination was quite limited (Figure 6). Similarly, ADE detection, information collection and assessment attracted most research efforts among the DS core activities, while signal detection, MoA analysis, and identification of drug interactions are the three most focused DS special topics. Contrariwise, the focus on ADE reporting is limited, and it can be identified as a gap for further research (Natsiavas et al., 2018).

Regarding the employed KE methods, NLP as well as graph-based inferencing were employed in many studies, while DL-based inferencing was quite limited. Interestingly, few studies exploited temporal modeling or analysis, despite the fact that time is extremely useful in the assessment of potential DS signals. In addition, very few studies employed holistic KE methodologies based e.g., on ontology patterns, quality control frameworks, etc. Adopting methodologies, such as MIRO (Matentzoglu et al., 2018), NeOn (Suárez-Figueroa et al., 2012), OQuaRE (Duque-Ramos et al., 2014), and XOD (He et al., 2018), could reinforce the credibility and the completeness of future contributions in the domain. Furthermore, the lack of focus on knowledge dissemination approaches is also evident.

Interestingly, some studies jointly exploited multiple data sources, illustrating, for example, the added value of KE methods regarding integration (Koutkias and Jaulent, 2016), as well as the interest for systematic linkage/modeling between the phenotype and elements of the genome/proteome that interact with the drug, and activated pathways to investigate the MoA. This need for a systematic approach facilitating the integration of low-level biochemical and genotypic information with phenotypic models applying the SP paradigm has been already identified as a research opportunity (He, 2016; Herrero-Zazo et al., 2016; Mager and Kimko, 2016). While such models illustrated remarkable results, they were not fully exploiting the power of ontologies, as they are typically based on rules (at least partially), in order to model physiological, biological, or pharmacodynamic/pharmacokinetic processes, and not using reference ontological models depicting Systems Biology or SP concepts in a systematic manner. Therefore, the holistic modeling of ADRs, combining the power of ontologies and DL reasoning with the mathematical or empirical models of pharmacokinetics and pharmacodynamics is a topic of open research. Such an approach could enable the integration of big data sources (via ontologies) with SP multi-scale models, to facilitate *Precision Medicine*. Well-promising results were obtained by combining statistical-based inference on report data and ontology-based modeling and inference upon ADR characteristics and categories (Xie et al., 2016b); thus, this approach enhanced with SP models shall be considered also as a research opportunity and further elaborated by future studies.

With respect to technical challenges, reasoning performance constitutes an important issue, especially when considering large-scale knowledge models. For example, in order to avoid multiple inheritance, OAE (a quite big, reference ontology in the domain) asserts only one parent term and allows the other parent term(s) to be obtained automatically by reasoning (Xie et al., 2016b). Another example of compromising knowledge modeling in the sake of performance is the case of DINTO (Herrero-Zazo et al., 2015), where the ontology had to be simplified in order to be processed by existing reasoners. Thus, performance issues in DL reasoning software may be considered as a bottleneck for the real-world adoption of complex/large ontology models.

In terms of evaluation, the results presented in many of the reviewed articles significantly depended on manual work (e.g., data curation, annotation, etc.), or they were obtained by engaging a small group of experts. In addition, many of the presented KE approaches were evaluated focusing on a narrower scope than the one presented as their main use case. Overall, shortcomings related to evaluation were the most frequent in the reviewed studies (Figure 9).

Besides weaknesses/challenges, some remarkable outcomes reported in the reviewed studies include:

1. An improvement in ADR prediction by exploiting biomolecular functional network data in the context of clinical trials (Huang et al., 2011).
2. An ontologized version of MedDRA which can facilitate grouping of ADRs that correspond to the same medical condition (Bousquet et al., 2014).
3. The successful incorporation of contextualized, medication safety related CDSSs in commercial products (an EHR and a Computerized Physician Order Entry (CPOE) system) (Koutkias et al., 2012).
4. A semantic interoperability platform automatically generating ADE reports from EHR data, aiming to address underreporting by clinicians (Declerck et al., 2015; Yuksel et al., 2016).
5. The successful identification of adverse drug effect mentions in Twitter with precision exceeding 85% and F1 exceeding 81% (Eshleman and Singh, 2016).
6. The automatic detection of two novel drug interactions involving cytochrome p450 (CYP3A4) and albumin as potential drug interaction proteins from DIO (Arikuma et al., 2008).
7. The conclusion that live attenuated influenza vaccines have lower chance of inducing Guillain-Barre Syndrome and paralysis than trivalent (killed) inactivated influenza vaccine (Sarntivijai et al., 2012).
8. The extraction of novel, ontology-assisted ADE profiles regarding the *M. bovis* strain Bacillus Calmette—Guerin (Xie et al., 2016b).
9. A Web analytics platform relying on ontological reasoning, facilitating clinicians to conduct comparative drug analyses based on advanced and user-friendly analytics regarding ADEs and contraindications (Lamy et al., 2017).
10. An ontology-supported methodology for reporting ADRs to regulatory agencies, demonstrating automated diagnosis confirmation (through standardization), and improvement in the reporting process (Courtot et al., 2014).

**Figure 10:**
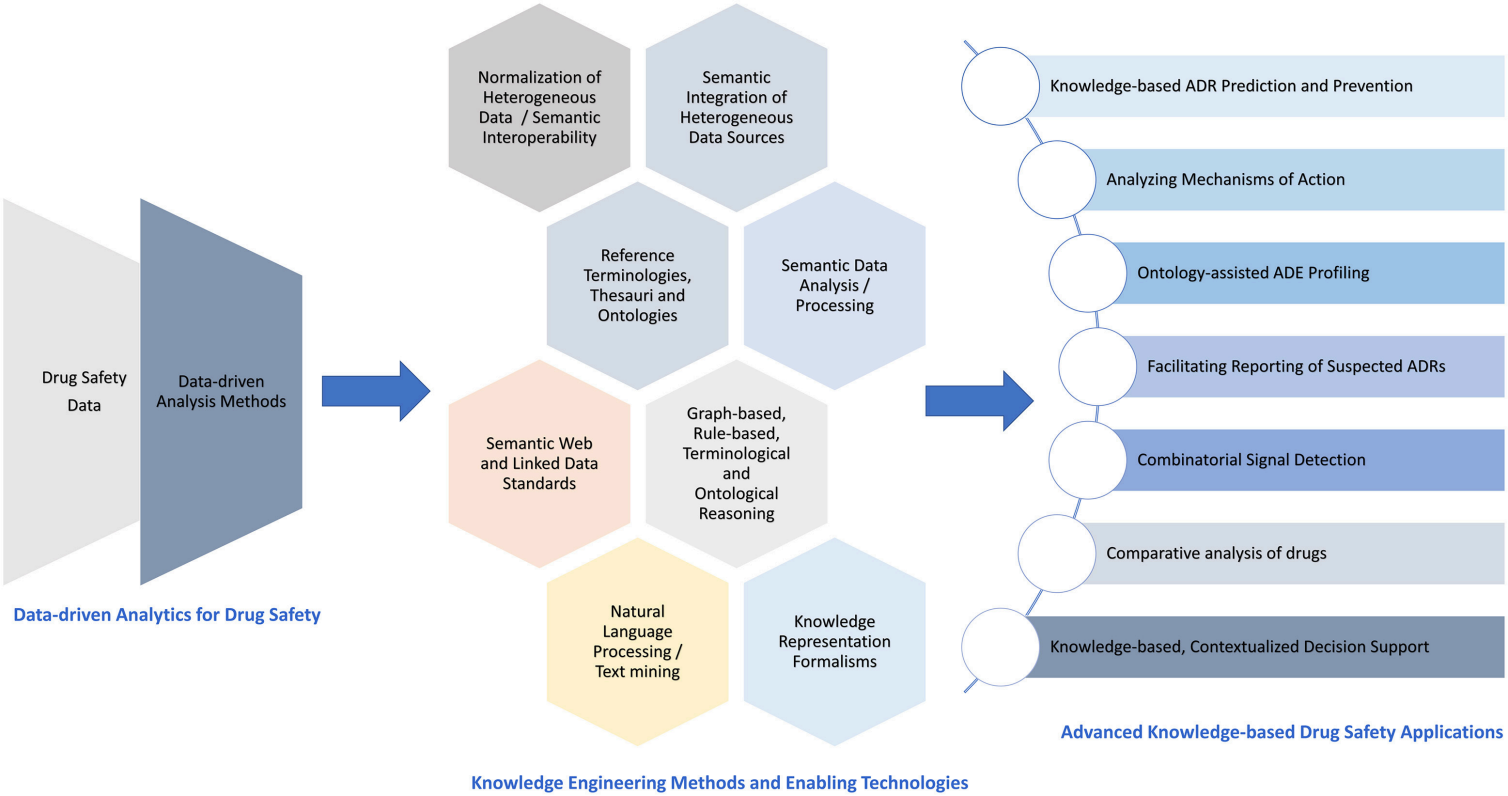
Advancing the data-driven perspective in DS through KE: methods, enabling technologies, and exemplar applications.

To this end, Figure 10 illustrates the identified advancements of the data-driven perspective in DS through KE with respect to methods, enabling technologies, and exemplar applications.

### Applications in Routine DS Practice

Employing ICT tools in routine DS practice conducted by hospitals, pharmaceutical companies, Contract Research Organizations (CROs) as well as drug regulatory organizations, imposes major challenges (Lu, 2009). In this subsection, we highlight the reviewed studies explicitly focusing on practical applications engaged with real-world environments as part of their pilot or validation phase.

The knowledge components of the CDSS developed in the PSIP project were based on EHR data obtained from three European countries (Koutkias et al., 2012). The CDSS was connected to the respective Hospital Information Systems (HIS) to identify and prevent potential ADEs. PSIP elaborated on contextualizing the CDSS knowledge in the particular local setting, such as the hospital/clinic and the targeted users. For example, in order to avoid over-alerting, alert generation was based on thresholds considering the statistical significance of each ADE rule in each particular clinical site. In addition, clinicians (both hospital pharmacists and medical doctors) were engaged in the system design and evaluation. The electronic service for DDI and ADE prevention during medication prescription of the Panacea CDSS was evaluated using patient data from a public hospital in Thessaloniki, Greece (Doulaverakis et al., 2014). Similarly, the E-pharmacovigilance system was deployed through a Web interface to present DS data to treating physicians (pediatric and internal medicine inpatient clinics from the University Hospital of Erlangen-Nurnberg) within the local HIS (Neubert et al., 2013).

The SALUS project focused on a practical implementation to automatically produce ADE reports based on real-world clinical data in two pilot sites (a regional clinical data warehouse maintained in Lombardy Region, Italy and the commercial EHR system in Uniklinikum Dresden, Germany) (Declerck et al., 2015). Notably, SALUS developed a signal analysis tool, which was validated by DS experts in pragmatic cases (Yuksel et al., 2016). Finally, a visual analytics platform to support comparative drug studies was deployed and evaluated by clinicians to assess the physician's decision whether to consider the new drug for future prescriptions (Lamy et al., 2017).

It is clear that most KE approaches are currently in an experimental phase and have not yet entered routine DS practice beyond pilots, due to technical challenges (e.g., automated reasoning upon big data volumes is computationally ineffective), or procedural/organizational challenges (e.g., need of clear evidence regarding CDSS performance, need to validate the respective knowledge data sources, etc.). The DS-related routine procedures which could be improved via KE-oriented applications can be summarized as follows: (a) DS procedures applied in the clinical environment to report and/or prevent ADEs, (b) DS-related information assessment and dissemination from the drug regulatory organizations point of view, and (c) assessment of potential signals in clinical trials of new drugs. The practical challenges of these processes in each context have already been highlighted. Notably, clinicians do not feel confident about the currently applied DS procedures (Vallano et al., 2015), while the need for better and more active DS surveillance has been identified by both drug regulatory organizations (Weaver et al., 2008) and the industry (Lu, 2009).

The use of KE technologies like the Semantic Web and Linked Data paradigms could significantly facilitate information and knowledge extraction, integration, elicitation and dissemination and, therefore, accommodate the imposed challenges on routine DS tasks. The main advantages of using KE approaches in real-world DS applications could be summarized as following:

- *Information linking* could be significantly improved and automated, reducing the need for manual data exploration through automatic processing.
- *Semantic enhancement* of already established information processing workflows (typically based on statistical measures like disproportionality analysis). The already established statistical processing could be combined with well-defined knowledge sources and their semantics to improve outcomes (e.g., regarding causality assessment).
- *Error prevention* could be facilitated by integrating different data sources to be used as control sources, in order to prevent false positives, over-alerting etc.
- *Process acceleration* as KE approaches could save a lot of time via (semi)-automatic data retrieval and interlinking.

The above advantages advocate for more intense research in the domain, in order to increase the level of maturity, which is necessary to employ KE approaches in routine DS practice.

### Limitations

The main source of limitation for the current study concerns the risk of bias. As explained in the Risk of bias subsection, our analysis considered such risks and followed specific mitigation actions to eliminate them. Subjectivity in the review process as well as in the definition of the domain is a significant issue for review studies. We believe that the participation of domain experts in our study, the employment of an appropriate conflict resolution protocol, as well as the adoption of reference definitions (e.g., to establish the analysis criteria) significantly reduced this issue. However, some risk inevitably remains; for example, in the paper submission phase we realized that one relevant paper was not included in our study (Bean et al., 2017), because we did not include the term “Knowledge Graph” (a term recently coined in the domain of KE) in our query terms. In addition, given that our query for article retrieval was focused on DS *per se*, some interesting resources that could be of wider scope, and not explicitly targeting or being used for DS, have not been considered in our study.

## Conclusions

Computational methods in the domain of DS have been primarily data-driven. However, in the era of big data, data heterogeneity and complexity hamper the application of these methods at large scale and across data sources. In order to overcome these shortcomings, an increasing number of studies employ KE methods and tools. Especially as semantic technologies and standards evolve and KE approaches gain awareness, the potential of enriching the traditional data analytics approaches for DS with knowledge-based components becomes stronger.

Along this perspective, this review highlighted exemplar research efforts by presenting a variety of knowledge-intensive activities applied in the DS field, such as normalization of DS data, integration of data from heterogeneous sources, the use of semantic models and terminologies to facilitate signal detection, and semantic processing of DS data, to name a few. In addition, we referred to a number of knowledge-based tools and platforms that have been employed to reinforce DS and support ADR detection, contextualized ADE prevention, and large-scale, semantically-enriched combinatorial signal detection. Through the conducted analysis, our study illustrated the contribution, the complementarity as well as the advances that KE-based approaches may bring to traditional data analytics applied in DS.

Despite the increasing number of studies exploiting KE for DS, the lack of a major “success-story”—beyond proof-of-concept—is apparent. This constitutes a significant challenge for researchers in the domain, that have to respond to, in order to foster the value of KE approaches in the context of DS and, therefore, facilitate their wider adoption by DS stakeholders. Although few studies reached or explicitly focused on routine DS practice, we argue that engaging KE methods in established DS processes can substantially contribute in the development of an advanced, continuous learning health system (Friedman et al., 2015), which is necessary for efficient DS surveillance. Finally, we suggest that the use of KE approaches, e.g., ontologies, in combination with *pharmacokinetics* and *pharmacodynamics* models could facilitate the construction of an SP framework able to provide a pathway toward *Precision Medicine* exploiting real-world evidence (Caudle et al., 2016; Helmlinger et al., 2017).

## Author Contributions

VK and M-CJ conceived the study in the scope of the SAFER project. VK supervised the study, defined the search protocol, and executed the queries against the two data sources (i.e., PubMed and Web of Science). PN and VK defined the inclusion/exclusion rules. PN, VK, and AM independently reviewed all the obtained articles, while CB and M-CJ contributed in conflict resolution. The analysis criteria were initially defined by VK, progressively refined by PN, and reviewed by AM, CB, and M-CJ. PN performed the data analysis and published the online interactive analytics graphs. AM provided expertise on biochemical/genetic data sources. All the authors contributed in the interpretation of the study findings and in writing the manuscript. All the authors reviewed and approved the content of the manuscript.

### Conflict of Interest Statement

The authors declare that the research was conducted in the absence of any commercial or financial relationships that could be construed as a potential conflict of interest.

## Footnotes

^1^Due to the numerous abbreviations used in the article, we provide as Appendix an abbreviation index to facilitate the reader.

^2^ADEs: Side-effects that may or may not have causal relationship with the drug, including the events caused by drug misuse (e.g., overdose). ADRs: Side-effects that occur after a legitimate drug use (i.e., there is no overdose) and therefore “characterized by the suspicion of a causal relationship between the drug and the occurrence.” For a comprehensive definition of terms used in DS we refer the reader to Lindquist (2007).

^3^By reviewing European studies (Formica et al., 2018) estimated: (a) the cost of ADRs between €2,851 and €9,015 for the inpatient setting and €174–8,515 for the outpatient setting; (b) the impact of ADRs on the length of stay to be 9.2 ± 0.2 days (outpatient setting) and 6.1 ± 2.3 days (inpatient setting). Furthermore, the US Office of Disease Prevention and Health Promotion estimated that ADEs account for 1 in 3 of all hospital adverse events, concern about 2 million hospital stays each year, and prolong hospital stays by 1.7–4.6 days. Regarding outpatient settings, each year ADEs account for over 3.5 million physician office visits, about 1 million emergency department visits, and ~125,000 hospital admissions (https://health.gov/hcq/ade.asp).

^4^“Information that arises from one or multiple sources (including observations and experiments) which suggests a new, potentially causal association, or a new aspect of a known association, between an intervention and an event or set of related events, either adverse or beneficial, that is judged to be of sufficient likelihood to justify verificatory action” (Council for International Organizations of Medical Sciences (CIOMS), 2010).

^5^An interesting review on text mining for ADEs has been presented by Harpaz et al. (2014).

^6^“In Computer and Information sciences, an ontology defines a set of representational primitives for modeling a domain of knowledge or discourse. The representational primitives are typically classes (or sets), attributes (or properties), and relationships (or relations among class members). The definitions of the representational primitives include information about their meaning and constraints on their logically consistent application” (Gruber, 2009).

^7^MedDRA® (the Medical Dictionary for Regulatory Activities) is a reference, international terminology in the domain of DS, developed under the auspices of the International Council for Harmonization of Technical Requirements for Pharmaceuticals for Human Use (ICH). The MedDRA® trademark is owned by IFPMA on behalf of ICH.

^8^A mapping review aims “*to map out and categorize existing literature on a particular topic, identifying gaps in research literature from which to commission further reviews and/or primary research*” (Grant and Booth, 2009).

^9^https://www.ncbi.nlm.nih.gov/pubmed/

^10^https://webofknowledge.com/

^11^https://pypi.org/project/BibReview/

^12^In such cases, only the most representative article was considered in our review.

^13^The detailed review results and the overall mapping outcome is documented in a spreadsheet, which is provided as Supplementary Material.

^14^The online analytics are available at: https://inab-certh.github.io/Knowledge-Engineering-for-Drug-Safety-Systematic-and-mapping-review/analytics and the respective files can be accessed at: https://github.com/inab-certh/Knowledge-Engineering-for-Drug-Safety-Systematic-and-mapping-review.

^15^The list of selected articles is provided as Supplementary Material.

^16^A catalog with Web links to data sources, reference terminologies, ontologies, standards, technologies, and systems referred in the study is provided as an Appendix.

^17^Only connections with weight >5 are depicted, to maintain readability.

^18^Not all referenced terminologies/ontologies are presented for readability purposes. The full list of referenced terminologies/ontologies can be found in the detailed analysis provided as Supplementary Material in the form of a spreadsheet.

^19^Not all data sources identified are presented for readability purposes. The full list of data sources identified can be found in the fully detailed analysis provided as Supplementary Material in the form of a spreadsheet.

^20^The special characteristics of each data source category and their possible contribution in signal detection are explicitly described in Koutkias and Jaulent (2015).

^21^In the detailed analysis results (provided as Supplementary Material), all referenced knowledge sources for each paper are identified. For readability, we only refer in the manuscript to the most prominent knowledge sources used.

^22^A relatively updated list of studies working on ADE extraction upon social media is presented in Liu et al. (2018), regardless if they employ KE techniques or not.

^23^https://www.ebi.ac.uk/rdf/

^24^http://www.openphactsfoundation.org

## Appendix

**Table A1:** Abbreviations used in the study.

**Abbreviation**	**Full text**
ADE	Adverse Drug Event
ADR	Adverse Drug Reaction
AUC	Area Under the receiver operating characteristic Curve
CDM	Common Data Model
CDSS	Clinical Decision Support System
CIG	Computer Interpretable Guidelines
CSCT	Case Series Characterization Tool
CIM	Common Information Model
CPOE	Computerized Physician Order Entry
DDI	Drug-drug interaction
DEI	Drug Enzyme Interaction ontology
DIO	Drug Interactions Ontology
DL	Description Logics
DS	Drug Safety
DSS	Decision Support Systems
EHR	Electronic Health Record
FDA	Food and Drug Administration
HCP	Healthcare Professional
HIS	Hospital Information System
IT	Information Technology
KE	Knowledge Engineering
ML	Machine Learning
MoA	Mechanism of Action
MPI	Multiple Pathway Interactions
NER	Named Entity Recognition
NLP	Natural Language Processing
PHR	Personal Health Record
PRR	Proportional Reporting Ratio
RRR	Relative Reporting Ratio
SNA	Social Network Analysis
SNP	Single Nucleotide Polymorphism
SP	Systems Pharmacology
SPL	Structured Product Label
SRS	Spontaneous Reporting System
TEO	Time Event Ontology
UMC	Uppsala Monitoring Centre
XOD	eXtensible Ontology Development

**Table A2:** Catalog with Web links to data sources, reference terminologies, ontologies, standards, technologies, and systems referred in the study.

**Abbreviation**	**Full text (if applicable) and link**
ADReCS	Adverse Drug Reaction Classification System (http://bioinf.xmu.edu.cn/ADReCS/)
ATC	Anatomical Therapeutic Chemical Classification system (https://www.whocc.no/atc_ddd_index/)
Bio2RDF	http://bio2rdf.org/
CheBI	Chemical Entities of Biological Interest (https://www.ebi.ac.uk/chebi/)
CTD	Comparative Toxicogenomics Database (http://ctdbase.org/)
D3	Drug-drug interaction Discovery and Demystification (https://scholar.colorado.edu/csci_gradetds/106/)
DAML + OIL	https://www.w3.org/TR/daml+oil-reference
DINTO	Drug-Drug Interactions Ontology (http://www.ontobee.org/ontology/DINTO)
DrOn	Drug Ontology (https://www.ebi.ac.uk/ols/ontologies/dron)
DrugBank	https://www.drugbank.ca/
E2B	http://www.ich.org/products/electronic-standards.html
FAERS	FDA Adverse Event Reporting System (https://www.fda.gov/drugs/guidancecomplianceregulatoryinformation/surveillance/adversedrugeffects/ucm082193.htm)
GO	Gene Ontology (http://www.geneontology.org/)
ICD	International Classification of Diseases (http://www.who.int/classifications/icd/en/)
ICT	Information and Communication Technologies
INDI	INferring Drug Interactions (https://www.cs.tau.ac.il/~bnet/software/INDI/)
KEGG	Kyoto Encyclopedia of Genes and Genomes—GenomeNet (https://www.genome.jp/kegg/)
LAERTES	Large-scale adverse effects related to treatment evidence standardization (https://github.com/OHDSI/KnowledgeBase/tree/master/LAERTES)
MedDRA	Medical Dictionary for Regulatory Activities (https://www.meddra.org/)
MeSH	Medical Subject Headings (https://www.nlm.nih.gov/mesh/)
MetaADEDB	http://lmmd.ecust.edu.cn/online_services/metaadedb/
MIRO	Minimal Information for Reporting of an Ontology (https://zenodo.org/record/398804)
NDF-RT	National Drug File—Reference Terminology (https://www.nlm.nih.gov/research/umls/sourcereleasedocs/current/NDFRT/)
OAE	Ontology for Adverse Events (http://www.oae-ontology.org/)
OCMR	Ontology of Chinese Medicine for Rheumatism (http://www.ontobee.org/ontology/OCMR)
OCVDAE	Ontology of Cardiovascular Drug Adverse Events (http://bioportal.bioontology.org/ontologies/OCVDAE)
ODNAE	Ontology of Drug Neuropathy Adverse Events (http://www.ontobee.org/ontology/ODNAE)
OGSF	Ontology of Genetic Susceptibility Factors (https://www.ebi.ac.uk/ols/ontologies/ogsf)
OHDSI	Observational Health Data Sciences and Informatics (https://www.ohdsi.org/)
OVAE	Ontology of Vaccine Adverse Events (http://www.violinet.org/ovae/)
OWL	Web Ontology Language (https://www.w3.org/OWL/)
OQuaRE	Ontology Quality Evaluation Framework (http://miuras.inf.um.es/oquarewiki/index.php5/Main_Page)
PharmGKB	Pharmacogenomics Knowledge Base (https://www.pharmgkb.org/)
PRISMA	Preferred Reporting Items for Systematic Reviews and Meta-Analyses (http://prisma-statement.org/)
PV-SDO	Pharmacovigilance Signal Detection Ontology (http://safer-project.eu/SignalDetectorsOntology.owl)
RDF	Resource Description Framework (https://www.w3.org/RDF/)
RxNorm	https://www.nlm.nih.gov/research/umls/rxnorm/
SemMedDB	Semantic MEDLINE Database (https://skr3.nlm.nih.gov/SemMedDB/)
SIDER	Side Effect Resource (http://sideeffects.embl.de/)
SMQ	Standardized MedDRA Queries (https://www.meddra.org/standardized-meddra-queries)
SNOMED-CT	Systematized Nomenclature of Medicine-Clinical Terms (https://www.snomed.org/)
SPARQL	SPARQL Protocol and RDF Query Language (https://www.w3.org/TR/2013/REC-sparql11-overview-20130321)
SWRL	Semantic Web Rules Language (https://www.w3.org/Submission/SWRL/)
UMLS	Unified Medical Language System (https://www.nlm.nih.gov/research/umls/)
UniProt	https://www.uniprot.org/
VAERS	Vaccine Adverse Event Reporting System (https://vaers.hhs.gov/)
VO	Vaccine Ontology (http://www.violinet.org/vaccineontology/)
WADM	Web Annotation Data Model (https://www.w3.org/TR/annotation-model/)

## References

[B1] Abdelaziz I. Hassanzadeh O. Zhang P. Sadoghi M. (2017). Large-scale structural and textual similarity-based mining of knowledge graph to predict drug–drug interactions. Web Semant. Sci. Serv. Agents World Wide Web 44, 104–117. 10.1016/J.WEBSEM.2017.06.002

[B2] Alecu I. Bousquet C. Jaulent M.-C. (2008). A case report: using SNOMED CT for grouping adverse drug reactions terms. BMC Med. Inform. Decis. Mak. 8 Suppl. 1:S4. 10.1186/1472-6947-8-S1-S4 19007441 PMC2582791

[B3] Altman D. Antes G. Gøtzsche P. Higgins J. Jüni P. Lewis S. (2011). Assessing risk of bias in included studies, in Cochrane Handbook for Systematic Reviews of Interventions Version 5.1.0, eds. Higgins J. P. Altman D. G. Sterne J. A. Available online at: www.handbook.cochrane.org.

[B4] Arikuma T. Yoshikawa S. Azuma R. Watanabe K. Matsumura K. Konagaya A. (2008). Drug interaction prediction using ontology-driven hypothetical assertion framework for pathway generation followed by numerical simulation. BMC Bioinformatics 9 Suppl. 6:S11. 10.1186/1471-2105-9-S6-S11 18541046 PMC2423434

[B5] Audeh B. Beigbeder M. Zimmermann A. Jaillon P. Bousquet C. (2017). Vigi4Med Scraper: a framework for web forum structured data extraction and semantic representation. PLoS ONE 12:e169658. 10.1371/journal.pone.0169658 28122056 PMC5266266

[B6] Baader F. Horrocks I. Sattler U. (2004). Description logics, in Handbook on Ontologies, eds. Staab S. Studer R. (Berlin; Heidelberg: Springer Berlin Heidelberg), 3–28. 10.1007/978-3-540-24750-0_1

[B7] Bai J. P. Fontana R. J. Price N. D. Sangar V. (2014). Systems pharmacology modeling: an approach to improving drug safety. Biopharm. Drug Dispos. 35, 1–14. 10.1002/bdd.1871 24136298

[B8] Bean D. M. Wu H. Iqbal E. Dzahini O. Ibrahim Z. M. Broadbent M.. (2017). Knowledge graph prediction of unknown adverse drug reactions and validation in electronic health records. Sci. Rep. 7:16416. 10.1038/s41598-017-16674-x 29180758 PMC5703951

[B9] Birtwistle M. R. Hansen J. Gallo J. M. Muppirisetty S. Ung P. M.-U. Iyengar R. (2016). Systems pharmacology: an overview, in Systems Pharmacology and Pharmacodynamics, eds. Mager D. E. Kimko H. H. C. (Cham: Springer), 53–80. 10.1007/978-3-319-44534-2_4

[B10] Bisgin H. Liu Z. Fang H. Xu X. Tong W. (2011). Mining FDA drug labels using an unsupervised learning technique–topic modeling. BMC Bioinformatics 12 Suppl. 10:S11. 10.1186/1471-2105-12-S10-S11 22166012 PMC3236833

[B11] Bjørnson F. O. Dingsøyr T. (2008). Knowledge management in software engineering: a systematic review of studied concepts, findings and research methods used. Inf. Softw. Technol. 50, 1055–1068. 10.1016/J.INFSOF.2008.03.006

[B12] Boland M. R. Jacunski A. Lorberbaum T. Romano J. D. Moskovitch R. Tatonetti N. P. (2016). Systems biology approaches for identifying adverse drug reactions and elucidating their underlying biological mechanisms. Wiley Interdiscip. Rev. Syst. Biol. Med. 8, 104–122. 10.1002/wsbm.1323 26559926 PMC4760887

[B13] Bousquet C. Henegar C. Louët A. L. Degoulet P. Jaulent M.-C. (2005a). Implementation of automated signal generation in pharmacovigilance using a knowledge-based approach. Int. J. Med. Inform. 74, 563–571. 10.1016/j.ijmedinf.2005.04.006 15955732

[B14] Bousquet C. Lagier G. Lillo-Le Louët A. Le Beller C. Venot A. Jaulent M.-C. (2005b). Appraisal of the MedDRA conceptual structure for describing and grouping adverse drug reactions. Drug Saf. 28, 19–34. 10.2165/00002018-200528010-00002 15649103

[B15] Bousquet C. Sadou É. Souvignet J. Jaulent M.-C. Declerck G. (2014). Formalizing MedDRA to support semantic reasoning on adverse drug reaction terms. J. Biomed. Inform. 49, 282–291. 10.1016/j.jbi.2014.03.012 24680984

[B16] Boyce R. D. Horn J. R. Hassanzadeh O. Waard A. de, Schneider J. Luciano J. S.. (2013). Dynamic enhancement of drug product labels to support drug safety, efficacy, and effectiveness. J. Biomed. Semantics 4, 5. 10.1186/2041-1480-4-5 23351881 PMC3698101

[B17] Boyce R. D. Ryan P. B. Norén G. N. Schuemie M. J. Reich C. Duke J.. (2014). Bridging islands of information to establish an integrated knowledge base of drugs and health outcomes of interest. Drug Saf. 37, 557–567. 10.1007/s40264-014-0189-0 24985530 PMC4134480

[B18] Bravo À. Li T. S. Su A. I. Good B. M. Furlong L. I. (2016). Combining machine learning, crowdsourcing and expert knowledge to detect chemical-induced diseases in text. Database (Oxford). 2016:baw94. 10.1093/database/baw094 27307137 PMC4908671

[B19] Cai M.-C. Xu Q. Pan Y.-J. Pan W. Ji N. Li Y.-B.. (2015). ADReCS: an ontology database for aiding standardization and hierarchical classification of adverse drug reaction terms. Nucleic Acids Res. 43, 907–913. 10.1093/nar/gku1066 25361966 PMC4383906

[B20] Cai R. Liu M. Hu Y. Melton B. L. Matheny M. E. Xu H.. (2017). Identification of adverse drug-drug interactions through causal association rule discovery from spontaneous adverse event reports. Artif. Intell. Med. 76, 7–15. 10.1016/j.artmed.2017.01.004 28363289 PMC6438384

[B21] Cañada A. Capella-Gutierrez S. Rabal O. Oyarzabal J. Valencia A. Krallinger M. (2017). LimTox: a web tool for applied text mining of adverse event and toxicity associations of compounds, drugs and genes. Nucleic Acids Res. 45, W484–W489. 10.1093/nar/gkx462 28531339 PMC5570141

[B22] Caudle K. E. Gammal R. S. Whirl-Carrillo M. Hoffman J. M. Relling M. V. Klein T. E. (2016). Evidence and resources to implement pharmacogenetic knowledge for precision medicine. Am. J. Health. Syst. Pharm. 73, 1977–1985. 10.2146/ajhp150977 27864205 PMC5117674

[B23] Ceusters W. Capolupo M. de Moor G. Devlies J. Smith B. (2011). An evolutionary approach to realism-based adverse event representations. Methods Inf. Med. 50, 62–73. 10.3414/ME10-02-0016 21057717 PMC3103706

[B24] Cheng F. Li W. Wang X. Zhou Y. Wu Z. Shen J.. (2013). Adverse drug events: database construction and *in silico* prediction. J. Chem. Inf. Model. 53, 744–752. 10.1021/ci4000079 23521697

[B25] Cheng F. Zhao Z. (2014). Machine learning-based prediction of drug-drug interactions by integrating drug phenotypic, therapeutic, chemical, and genomic properties. J. Am. Med. Inform. Assoc. 21, 278–286. 10.1136/amiajnl-2013-002512 24644270 PMC4173180

[B26] Cocos A. Fiks A. G. Masino A. J. (2017). Deep learning for pharmacovigilance: recurrent neural network architectures for labeling adverse drug reactions in Twitter posts. J. Am. Med. Informatics Assoc. 24, 813–821. 10.1093/jamia/ocw180 28339747 PMC7651964

[B27] Cohen T. Widdows D. (2017). Embedding of semantic predications. J. Biomed. Inform. 68, 150–166. 10.1016/j.jbi.2017.03.003 28284761 PMC5441848

[B28] Collins F. S. Varmus H. (2015). A new initiative on precision medicine. N. Engl. J. Med. 372, 793–795. 10.1056/NEJMp1500523 25635347 PMC5101938

[B29] Council for International Organizations of Medical Sciences (CIOMS) (2010). Practical Aspects of Signal Detection in Pharmacovigilance, Council for International Organizations of Medical Sciences. Report of CIOMS Working Group VIII. 1st Edn. Geneva: CIOMS Available online at: http://www.cioms.ch/index.php/publications/available-publications/540/view/54/other/27/practical-aspects-of-signal-detection-in-pharmacovigilance-report-of-cioms-working-group-viii

[B30] Courtot M. Brinkman R. R. Ruttenberg A. (2014). The logic of surveillance guidelines: an analysis of vaccine adverse event reports from an ontological perspective. PLoS ONE 9:e92632. 10.1371/journal.pone.0092632 24667848 PMC3965435

[B31] Declerck G. Hussain S. Daniel C. Yuksel M. Laleci G. B. Twagirumukiza M.. (2015). Bridging data models and terminologies to support adverse drug event reporting using EHR data. Methods Inf. Med. 54, 24–31. 10.3414/ME13-02-0025 25487120

[B32] Doulaverakis C. Nikolaidis G. Kleontas A. Kompatsiaris I. (2014). Panacea, a semantic-enabled drug recommendations discovery framework. J. Biomed. Semantics 5:13. 10.1186/2041-1480-5-13 24602515 PMC3975719

[B33] Drucker A. M. Fleming P. Chan A.-W. (2016). Research techniques made simple: assessing risk of bias in systematic reviews. J. Invest. Dermatol. 136, 109–114. 10.1016/J.JID.2016.08.021 27772550

[B34] Dupuch M. Grabar N. (2015). Semantic distance-based creation of clusters of pharmacovigilance terms and their evaluation. J. Biomed. Inform. 54, 174–185. 10.1016/J.JBI.2014.11.007 25659451

[B35] Duque-Ramos A. Boeker M. Jansen L. Schulz S. Iniesta M. Fernández-Breis J. T. (2014). Evaluating the Good Ontology Design Guideline (GoodOD) with the ontology quality requirements and evaluation method and metrics (OQuaRE). PLoS ONE 9:e104463. 10.1371/journal.pone.0104463 25148262 PMC4141745

[B36] Eshleman R. Singh R. (2016). Leveraging graph topology and semantic context for pharmacovigilance through Twitter-streams. BMC Bioinformatics 17:335. 10.1186/s12859-016-1220-5 27766937 PMC5073861

[B37] Formica D. Sultana J. Cutroneo P. M. Lucchesi S. Angelica R. Crisafulli S.. (2018). The economic burden of preventable adverse drug reactions: a systematic review of observational studies. Expert Opin. Drug Saf. 17, 681–695. 10.1080/14740338.2018.1491547 29952667

[B38] Fox J. (1984). A short account of knowledge engineering. Knowl. Eng. Rev. 1:4 10.1017/S0269888900000424

[B39] Friedman C. Rubin J. Brown J. Buntin M. Corn M. Etheredge L.. (2015). Toward a science of learning systems: a research agenda for the high-functioning Learning Health System. J. Am. Med. Inform. Assoc. 22, 43–50. 10.1136/amiajnl-2014-002977 25342177 PMC4433378

[B40] Gottlieb A. Stein G. Y. Oron Y. Ruppin E. Sharan R. (2012). INDI: a computational framework for inferring drug interactions and their associated recommendations. Mol. Syst. Biol. 8, 592. 10.1038/msb.2012.26 22806140 PMC3421442

[B41] Grant M. J. Booth A. (2009). A typology of reviews: an analysis of 14 review types and associated methodologies. Health Info. Libr. J. 26, 91–108. 10.1111/j.1471-1842.2009.00848.x 19490148

[B42] Gruber T. (2009). Ontology, in Encyclopedia of Database Systems, eds. Ling Liu Özsu M. T. (Boston, MA: Springer US), 1963–1965. 10.1007/978-0-387-39940-9_1318

[B43] Guo A. Racz R. Hur J. Lin Y. Xiang Z. Zhao L.. (2016). Ontology-based collection, representation and analysis of drug-associated neuropathy adverse events. J. Biomed. Semantics 7:29. 10.1186/s13326-016-0069-x 27213033 PMC4875649

[B44] Gurulingappa H. Mateen-Rajput A. Toldo L. (2012). Extraction of potential adverse drug events from medical case reports. J. Biomed. Semantics 3, 15. 10.1186/2041-1480-3-15 23256479 PMC3599676

[B45] Harpaz R. Callahan A. Tamang S. Low Y. Odgers D. Finlayson S.. (2014). Text mining for adverse drug events: the promise, challenges, and state of the art. Drug Saf. 37, 777–790. 10.1007/s40264-014-0218-z 25151493 PMC4217510

[B46] Harpaz R. DuMouchel W. Shah N. H. Madigan D. Ryan P. Friedman C. (2012). Novel data mining methodologies for adverse drug event discovery and analysis. Clin. Pharmacol. Ther. 91, 1010–1021. 10.1038/clpt.2012.50 22549283 PMC3675775

[B47] Hauben M. Norén G. N. (2010). A decade of data mining and still counting. Drug Saf. 33, 527–534. 10.2165/11532430-000000000-00000 20553054

[B48] He L. Yang Z. Zhao Z. Lin H. Li Y. (2013). Extracting drug-drug interaction from the biomedical literature using a stacked generalization-based approach. PLoS ONE 8:e65814. 10.1371/journal.pone.0065814 23785452 PMC3681788

[B49] He Y. (2016). Ontology-based vaccine and drug adverse event representation and theory-guided systematic causal network analysis toward integrative pharmacovigilance research. Curr. Pharmacol. Rep. 2, 113–128. 10.1007/s40495-016-0055-0 27458549 PMC4957564

[B50] He Y. Racz R. Sayers S. Lin Y. Todd T. Hur J.. (2014a). Updates on the web-based VIOLIN vaccine database and analysis system. Nucleic Acids Res. 42, D1124–D1132. 10.1093/nar/gkt1133 24259431 PMC3964998

[B51] He Y. Sarntivijai S. Lin Y. Xiang Z. Guo A. Zhang S.. (2014b). OAE: the ontology of adverse events. J. Biomed. Semantics 5:29. 10.1186/2041-1480-5-29 25093068 PMC4120740

[B52] He Y. Xiang Z. Zheng J. Lin Y. Overton J. A. Ong E. (2018). The eXtensible ontology development (XOD) principles and tool implementation to support ontology interoperability. J. Biomed. Semantics 9:3. 10.1186/s13326-017-0169-2 29329592 PMC5765662

[B53] Helmlinger G. Al-Huniti N. Aksenov S. Peskov K. Hallow K. M. Chu L. (2017). Drug-disease modeling in the pharmaceutical industry–where mechanistic systems pharmacology and statistical pharmacometrics meet. Eur. J. Pharm. Sci. 109, S39–S46. 10.1016/J.EJPS.2017.05.028 28506868

[B54] Henegar C. Bousquet C. Lillo-Le Louët A. Degoulet P. Jaulent M.-C. (2006). Building an ontology of adverse drug reactions for automated signal generation in pharmacovigilance. Comput. Biol. Med. 36, 748–767. 10.1016/j.compbiomed.2005.04.009 16185681

[B55] Henriksson A. Kvist M. Dalianis H. Duneld M. (2015). Identifying adverse drug event information in clinical notes with distributional semantic representations of context. J. Biomed. Inform. 57, 333–349. 10.1016/j.jbi.2015.08.013 26291578

[B56] Henriksson A. Zhao J. Dalianis H. Boström H. (2016). Ensembles of randomized trees using diverse distributed representations of clinical events. BMC Med. Inform. Decis. Mak. 16 Suppl. 2:69 10.1186/s12911-016-0309-0 PMC4965720 27459846

[B57] Herrero-Zazo M. Segura-Bedmar I. Hastings J. Martínez P. (2015). DINTO: using OWL ontologies and SWRL rules to infer drug–drug interactions and their mechanisms. J. Chem. Inf. Model. 55, 1698–1707. 10.1021/acs.jcim.5b00119 26147071

[B58] Herrero-Zazo M. Segura-Bedmar I. Martínez P. (2016). Conceptual models of drug-drug interactions: A summary of recent efforts. Knowl. Based Syst. 114, 99–107. 10.1016/j.knosys.2016.10.006

[B59] Hogan W. R. Hanna J. Hicks A. Amirova S. Bramblett B. Diller M.. (2017). Therapeutic indications and other use-case-driven updates in the drug ontology: anti-malarials, anti-hypertensives, opioid analgesics, and a large term request. J. Biomed. Semantics 8:10. 10.1186/s13326-017-0121-5 28253937 PMC5335794

[B60] Huang L.-C. Wu X. Chen J. Y. (2011). Predicting adverse side effects of drugs. BMC Genomics 12 Suppl. 5:S11 10.1186/1471-2164-12-S5-S11 PMC3287493 22369493

[B61] Hur J. Ozgür A. Xiang Z. He Y. (2012). Identification of fever and vaccine-associated gene interaction networks using ontology-based literature mining. J. Biomed. Semantics 3:18. 10.1186/2041-1480-3-18 23256563 PMC3599673

[B62] Iqbal E. Mallah R. Rhodes D. Wu H. Romero A. Chang N.. (2017). ADEPt, a semantically-enriched pipeline for extracting adverse drug events from free-text electronic health records. PLoS ONE 12:e0187121. 10.1371/journal.pone.0187121 29121053 PMC5679515

[B63] Iyer S. V. Harpaz R. LePendu P. Bauer-Mehren A. Shah N. H. (2014). Mining clinical text for signals of adverse drug-drug interactions. J. Am. Med. Informatics Assoc. 21, 353–362. 10.1136/amiajnl-2013-001612 24158091 PMC3932451

[B64] Jiang G. Liu H. Solbrig H. R. Chute C. G. (2015). Mining severe drug-drug interaction adverse events using Semantic Web technologies: a case study. BioData Min. 8:12. 10.1186/s13040-015-0044-6 25829948 PMC4379609

[B65] Kang N. Singh B. Bui C. Afzal Z. van Mulligen E. M. Kors J. A. (2014). Knowledge-based extraction of adverse drug events from biomedical text. BMC Bioinformatics 15:64. 10.1186/1471-2105-15-64 24593054 PMC3973995

[B66] Kawazoe Y. Imai T. Ohe K. (2016). A querying method over RDF-ized Health Level Seven v2.5 messages using life science knowledge resources. JMIR Med. Informatics 4:e12. 10.2196/medinform.5275 27050304 PMC4837294

[B67] Kilicoglu H. Shin D. Fiszman M. Rosemblat G. Rindflesch T. C. (2012). SemMedDB: a PubMed-scale repository of biomedical semantic predications. Bioinformatics 28, 3158–3160. 10.1093/bioinformatics/bts591 23044550 PMC3509487

[B68] Klein A. Z. Sarker A. Cai H. Weissenbacher D. Gonzalez-Hernandez G. (2018). Social media mining for birth defects research: a rule-based, bootstrapping approach to collecting data for rare health-related events on Twitter. J. Biomed. Inform. 87, 68–78. 10.1016/J.JBI.2018.10.001 30292855 PMC6295660

[B69] Knowledge Base Workgroup of the Observational Health Data Sciences and Informatics (OHDSI) Collaborative (2017). Large-scale adverse effects related to treatment evidence standardization (LAERTES): an open scalable system for linking pharmacovigilance evidence sources with clinical data. J. Biomed. Semantics 8:11 10.1186/s13326-017-0115-3 28270198 PMC5341176

[B70] Koutkias V. (2019). From data silos to standardized, linked, and FAIR data for pharmacovigilance: current advances and challenges with observational healthcare data. Drug Saf. 42, 583–586. 10.1007/s40264-018-00793-z 30666591

[B71] Koutkias V. Jaulent M.-C. (2016). A multiagent system for integrated detection of pharmacovigilance signals. J. Med. Syst. 40, 37. 10.1007/s10916-015-0378-0 26590975

[B72] Koutkias V. Kilintzis V. Stalidis G. Lazou K. Niès J. Durand-Texte L.. (2012). Knowledge engineering for adverse drug event prevention: On the design and development of a uniform, contextualized and sustainable knowledge-based framework. J. Biomed. Inform. 45, 495–506. 10.1016/j.jbi.2012.01.007 22326287

[B73] Koutkias V. G. Jaulent M.-C. (2015). Computational approaches for pharmacovigilance signal detection: toward integrated and semantically-enriched frameworks. Drug Saf. 38, 219–232. 10.1007/s40264-015-0278-8 25749722 PMC4374117

[B74] Koutkias V. G. Lillo-Le Louët A. Jaulent M.-C. (2017). Exploiting heterogeneous publicly available data sources for drug safety surveillance: computational framework and case studies. Expert Opin. Drug Saf. 16, 113–124. 10.1080/14740338.2017.1257604 27813420

[B75] Lamy J.-B. Berthelot H. Favre M. Ugon A. Duclos C. Venot A. (2017). Using visual analytics for presenting comparative information on new drugs. J. Biomed. Inform. 71, 58–69. 10.1016/J.JBI.2017.04.019 28549568

[B76] Lamy J.-B. Séroussi B. Griffon N. Kerdelhué G. Jaulent M.-C. Bouaud J. (2015). Toward a formalization of the process to select IMIA Yearbook best papers. Methods Inf. Med. 54, 135–144. 10.3414/ME14-01-0031 25396220

[B77] Lin S.-F. Xiao K.-T. Huang Y.-T. Chiu C.-C. Soo V.-W. (2010). Analysis of adverse drug reactions using drug and drug target interactions and graph-based methods. Artif. Intell. Med. 48, 161–166. 10.1016/j.artmed.2009.11.002 19962282

[B78] Lin Y. He Y. (2012). Ontology representation and analysis of vaccine formulation and administration and their effects on vaccine immune responses. J. Biomed. Semantics 3:17. 10.1186/2041-1480-3-17 23256535 PMC3639077

[B79] Lin Y. He Y. (2014). The ontology of genetic susceptibility factors (OGSF) and its application in modeling genetic susceptibility to vaccine adverse events. J. Biomed. Semantics 5:19. 10.1186/2041-1480-5-19 24963371 PMC4068904

[B80] Lindquist M. (2007). The need for definitions in pharmacovigilance. Drug Saf. 30, 825–830. 10.2165/00002018-200730100-00001 17867720

[B81] Liu J. Zhao S. Wang G. (2018). SSEL-ADE: a semi-supervised ensemble learning framework for extracting adverse drug events from social media. Artif. Intell. Med. 84, 34–49. 10.1016/J.ARTMED.2017.10.003 29111222

[B82] Liu J. Zhao S. Zhang X. (2016). An ensemble method for extracting adverse drug events from social media. Artif. Intell. Med. 70, 62–76. 10.1016/J.ARTMED.2016.05.004 27431037

[B83] Liu Q. Wang J. Zhu Y. He Y. (2017). Ontology-based systematic representation and analysis of traditional Chinese drugs against rheumatism. BMC Syst. Biol. 11:130. 10.1186/s12918-017-0510-5 29322929 PMC5763303

[B84] Liu X. Chen H. (2015). A research framework for pharmacovigilance in health social media: Identification and evaluation of patient adverse drug event reports. J. Biomed. Inform. 58, 268–279. 10.1016/j.jbi.2015.10.011 26518315

[B85] Lorberbaum T. Nasir M. Keiser M. J. Vilar S. Hripcsak G. Tatonetti N. P. (2015). Systems pharmacology augments drug safety surveillance. Clin. Pharmacol. Ther. 97, 151–158. 10.1002/cpt.2 25670520 PMC4325423

[B86] Lowe D. M. O'Boyle N. M. Sayle R. A. (2016). Efficient chemical-disease identification and relationship extraction using Wikipedia to improve recall. Database (Oxford). 2016:baw039. 10.1093/database/baw039 27060160 PMC4825350

[B87] Lu Z. (2009). Information technology in pharmacovigilance: benefits, challenges, and future directions from industry perspectives. Drug. Healthc. Patient Saf. 1, 35–45. 10.2147/DHPS.S7180 21701609 PMC3108683

[B88] Mager D. E. Kimko H. H. C. (2016). Systems Pharmacology and Pharmacodynamics. Cham: Springer International Publishing 10.1007/978-3-319-44534-2

[B89] Marcos E. Zhao B. He Y. (2013). The Ontology of Vaccine Adverse Events (OVAE) and its usage in representing and analyzing adverse events associated with US-licensed human vaccines. J. Biomed. Semantics 4:40. 10.1186/2041-1480-4-40 24279920 PMC4177204

[B90] Matentzoglu N. Malone J. Mungall C. Stevens R. (2018). MIRO: guidelines for minimum information for the reporting of an ontology. J. Biomed. Semantics 9:6. 10.1186/s13326-017-0172-7 29347969 PMC5774126

[B91] Moher D. Liberati A. Tetzlaff J. Altman D. G. Group T. P. (2009). Preferred reporting items for systematic reviews and meta-analyses: the PRISMA statement. PLoS Med. 6:e1000097 10.1371/journal.pmed.1000097 19621072 PMC2707599

[B92] Montastruc J.-L. Sommet A. Bagheri H. Lapeyre-Mestre M. (2011). Benefits and strengths of the disproportionality analysis for identification of adverse drug reactions in a pharmacovigilance database. Br. J. Clin. Pharmacol. 72, 905–908. 10.1111/j.1365-2125.2011.04037.x 21658092 PMC3244636

[B93] Natsiavas P. Boyce R. D. Jaulent M.-C. Koutkias V. (2018). OpenPVSignal: advancing information search, sharing and reuse on pharmacovigilance signals via FAIR principles and Semantic Web technologies. Front. Pharmacol. 9:609. 10.3389/fphar.2018.00609 29997499 PMC6028717

[B94] Neubert A. Dormann H. Prokosch H.-U. Bürkle T. Rascher W. Sojer R. (2013). E-pharmacovigilance: development and implementation of a computable knowledge base to identify adverse drug reactions. Br. J. Clin. Pharmacol. 76 Suppl. 1, 69–77. 10.1111/bcp.12127 PMC3781681 23586589

[B95] Nguyen T. Larsen M. E. O'Dea B. Phung D. Venkatesh S. Christensen H. (2017). Estimation of the prevalence of adverse drug reactions from social media. Int. J. Med. Inform. 102, 130–137. 10.1016/j.ijmedinf.2017.03.013 28495341

[B96] Nikfarjam A. Sarker A. O'Connor K. Ginn R. Gonzalez G. (2015). Pharmacovigilance from social media: mining adverse drug reaction mentions using sequence labeling with word embedding cluster features. J. Am. Med. Inform. Assoc. 22, 671–681. 10.1093/jamia/ocu041 25755127 PMC4457113

[B97] Noor A. Assiri A. Ayvaz S. Clark C. Dumontier M. (2016). Drug-drug interaction discovery and demystification using Semantic Web technologies. J. Am. Med. Informatics Assoc., ocw128. 10.1093/jamia/ocw128 28031284 PMC7651897

[B98] Pappalardo F. Russo G. Tshinanu F. M. Viceconti M. (2018). In silico clinical trials: concepts and early adoptions *Brief. Bioinform* 10.1093/bib/bby043. [Epub ahead of print]. 29868882

[B99] Personeni G. Bresso E. Devignes M.-D. Dumontier M. Smaïl-Tabbone M. Coulet A. (2017). Discovering associations between adverse drug events using pattern structures and ontologies. J. Biomed. Semantics 8:29. 10.1186/s13326-017-0137-x 28830518 PMC5567667

[B100] Piñero J. Bravo À. Queralt-Rosinach N. Gutiérrez-Sacristán A. Deu-Pons J. Centeno E.. (2017). DisGeNET: a comprehensive platform integrating information on human disease-associated genes and variants. Nucleic Acids Res. 45, D833–D839. 10.1093/nar/gkw943 27924018 PMC5210640

[B101] Queralt-Rosinach N. Piñero J. Bravo À. Sanz F. Furlong L. I. (2016). DisGeNET-RDF: harnessing the innovative power of the Semantic Web to explore the genetic basis of diseases. Bioinformatics 32, 2236–2238. 10.1093/bioinformatics/btw214 27153650 PMC4937199

[B102] Ramanujan S. Gadkar K. Kadambi A. (2016). Quantitative systems pharmacology: applications and adoption in drug development, in Systems Pharmacology and Pharmacodynamics, eds. Mager D. E. Kimko H. H. C. (Cham: Springer), 27–52. 10.1007/978-3-319-44534-2_3

[B103] Rieger T. R. Allen R. J. Bystricky L. Chen Y. Colopy G. W. Cui Y.. (2018). Improving the generation and selection of virtual populations in quantitative systems pharmacology models. Prog. Biophys. Mol. Biol. 139, 15–22. 10.1016/J.PBIOMOLBIO.2018.06.002 29902482

[B104] Sarker A. Gonzalez G. (2015). Portable automatic text classification for adverse drug reaction detection via multi-corpus training. J. Biomed. Inform. 53, 196–207. 10.1016/j.jbi.2014.11.002 25451103 PMC4355323

[B105] Sarntivijai S. Xiang Z. Shedden K. A. Markel H. Omenn G. S. Athey B. D.. (2012). Ontology-based combinatorial comparative analysis of adverse events associated with killed and live influenza vaccines. PLoS ONE 7:e49941. 10.1371/journal.pone.0049941 23209624 PMC3509157

[B106] Sarntivijai S. Zhang S. Jagannathan D. G. Zaman S. Burkhart K. K. Omenn G. S.. (2016). Linking MedDRA^®^-coded clinical phenotypes to biological mechanisms by the ontology of adverse events: a pilot study on tyrosine kinase inhibitors. Drug Saf. 39, 697–707. 10.1007/s40264-016-0414-0 27003817 PMC4933310

[B107] Schotland P. Bojunga N. Zien A. Trame M. N. Lesko L. J. (2016). Improving drug safety with a systems pharmacology approach. Eur. J. Pharm. Sci. 94, 84–92. 10.1016/J.EJPS.2016.06.009 27287422

[B108] Schreiber G. (2008). Knowledge engineering, in Handbook of Knowledge Representation, eds. Van Harmelen F. Lifschitz V. Porter B. (Elsevier), 929–946. 10.1016/S1574-6526(07)03025-8

[B109] Segura-Bedmar I. Crespo M. de Pablo-Sánchez C. Martínez P. (2010). Resolving anaphoras for the extraction of drug-drug interactions in pharmacological documents. BMC Bioinformatics 11 Suppl. 2:S1 10.1186/1471-2105-11-S2-S1 PMC3288782 20406499

[B110] Segura-Bedmar I. Martínez P. (2017). Simplifying drug package leaflets written in Spanish by using word embedding. J. Biomed. Semantics 8:45. 10.1186/s13326-017-0156-7 28962645 PMC5622567

[B111] Segura-Bedmar I. Martínez P. de Pablo-Sánchez C. (2011). A linguistic rule-based approach to extract drug-drug interactions from pharmacological documents. BMC Bioinformatics 12 Suppl. 2:S1 10.1186/1471-2105-12-S2-S1 PMC3073181 21489220

[B112] Shang N. Xu H. Rindflesch T. C. Cohen T. (2014). Identifying plausible adverse drug reactions using knowledge extracted from the literature. J. Biomed. Inform. 52, 293–310. 10.1016/j.jbi.2014.07.011 25046831 PMC4261011

[B113] Sinha V. Huang S.-M. Abernethy D. R. Wang Y. Zhao P. Zineh I. (2016). Role of systems modeling in regulatory drug approval, in Systems Pharmacology and Pharmacodynamics, eds. Mager D. E. Kimko H. H. C. (Cham: Springer), 15–25. 10.1007/978-3-319-44534-2_2

[B114] Souvignet J. Declerck G. Asfari H. Jaulent M.-C. Bousquet C. (2016). OntoADR a semantic resource describing adverse drug reactions to support searching, coding, and information retrieval. J. Biomed. Inform. 63, 100–107. 10.1016/j.jbi.2016.06.010 27369567

[B115] Suárez-Figueroa M. C. Gómez-Pérez A. Fernández-López M. (2012). The NeOn methodology for ontology engineering, in Ontology Engineering in a Networked World, eds. Suárez-Figueroa M. C. Gómez-Pérez A. Motta E. Gangemi A. (Berlin; Heidelberg: Springer Berlin Heidelberg), 9–34. 10.1007/978-3-642-24794-1_2

[B116] Tao C. He Y. Yang H. Poland G. A. Chute C. G. (2012). Ontology-based time information representation of vaccine adverse events in VAERS for temporal analysis. J. Biomed. Semantics 3:13. 10.1186/2041-1480-3-13 23256916 PMC3554604

[B117] Tari L. Anwar S. Liang S. Cai J. Baral C. (2010). Discovering drug-drug interactions: a text-mining and reasoning approach based on properties of drug metabolism. Bioinformatics 26, i547–i553. 10.1093/bioinformatics/btq382 20823320 PMC2935409

[B118] Trame M. N. Biliouris K. Lesko L. J. Mettetal J. T. (2016). Systems pharmacology to predict drug safety in drug development. Eur. J. Pharm. Sci. 94, 93–95. 10.1016/J.EJPS.2016.05.027 27251780

[B119] Vallano A. Castaneda P. Quijada Manuitt M. Simon P. Pedrós C Quintana B (2015). Hospital doctors' views and concerns about pharmacovigilance. J. Pharmacovigil. 3, 1–5. 10.4172/2329-6887.1000160

[B120] Vandervalk B. McCarthy E. L. Cruz-Toledo J. Klein A. Baker C. J. Dumontier M.. (2013). The SADI personal health lens: a web browser-based system for identifying personally relevant drug interactions. JMIR Res. Protoc. 2:e14. 10.2196/resprot.2315 23612187 PMC3628156

[B121] Voss E. A. Boyce R. D. Ryan P. B. van der Lei J. Rijnbeek P. R. Schuemie M. J. (2017). Accuracy of an automated knowledge base for identifying drug adverse reactions. J. Biomed. Inform. 66, 72–81. 10.1016/J.JBI.2016.12.005 27993747 PMC5316295

[B122] Voss E. A. Makadia R. Matcho A. Ma Q. Knoll C. Schuemie M.. (2015). Feasibility and utility of applications of the common data model to multiple, disparate observational health databases. J. Am. Med. Inform. Assoc. 22, 553–564. 10.1093/jamia/ocu023 25670757 PMC4457111

[B123] Wang L. Jiang G. Li D. Liu H. (2014). Standardizing adverse drug event reporting data. J. Biomed. Semantics 5. 10.1186/2041-1480-5-36 PMC4142531 25157320

[B124] Wang L. Li M. Xie J. Cao Y. Liu H. He Y. (2017). Ontology-based systematical representation and drug class effect analysis of package insert-reported adverse events associated with cardiovascular drugs used in China. Nat. Sci. Rep. 7:13819. 10.1038/s41598-017-12580-4 29061976 PMC5653862

[B125] Weaver J. Willy M. Avigan M. (2008). Informatic tools and approaches in postmarketing pharmacovigilance used by FDA. AAPS J. 10, 35–41. 10.1208/s12248-007-9004-5 18446503 PMC2751449

[B126] Wnuk K. Garrepalli T. (2018). Knowledge management in software testing: a systematic snowball literature review. eInformatica Softw. Eng. J. 12, 51–78. 10.5277/E-INF180103

[B127] World Health Organization (2008). A Practical Handbook on the Pharmacovigilance of Antimalarial Medicines. World Health Organization. Available online at: http://www.who.int/malaria/publications/atoz/9789241547499/en/ (accessed December 12, 2018).

[B128] World Health Organization W. C. C. for I. D. M. (2002). The Importance of Pharmacovigilance. World Health Organization. Available online at: http://apps.who.int/medicinedocs/en/d/Js4893e/ (accessed December 12, 2018).

[B129] Xie J. Codd C. Mo K. He Y. (2016a). Differential adverse event profiles associated with BCG as a preventive tuberculosis vaccine or therapeutic bladder cancer vaccine identified by comparative ontology-based VAERS and literature meta-analysis. PLoS ONE 11:e0164792. 10.1371/journal.pone.0164792 27749923 PMC5066964

[B130] Xie J. He Y. (2017). Ontology-based vaccine adverse event representation and analysis. Adv. Exp. Med. Biol. 1028, 89–103. 10.1007/978-981-10-6041-0_6 29058218

[B131] Xie J. Zhao L. Zhou S. He Y. (2016b). Statistical and ontological analysis of adverse events associated with monovalent and combination vaccines against hepatitis A and B diseases. Sci. Rep. 6:34318. 10.1038/srep34318 27694888 PMC5046117

[B132] Xu R. Wang Q. (2013). A semi-supervised approach to extract pharmacogenomics-specific drug-gene pairs from biomedical literature for personalized medicine. J. Biomed. Inform. 46, 585–593. 10.1016/j.jbi.2013.04.001 23570835 PMC4452014

[B133] Yokotsuka M. Aoyama M. Kubota K. (2000). The use of a medical dictionary for regulatory activities terminology (MedDRA) in prescription-event monitoring in Japan (J-PEM). Int. J. Med. Inform. 57, 139–153. 10.1016/S1386-5056(00)00062-9 10961570

[B134] Yuksel M. Gonul S. Laleci Erturkmen G. B. Sinaci A. A. Invernizzi P. Facchinetti S.. (2016). An interoperability platform enabling reuse of electronic health records for signal verification studies. Biomed Res. Int. 2016:6741418. 10.1155/2016/6741418 27123451 PMC4830705

[B135] Zhang R. Cairelli M. J. Fiszman M. Rosemblat G. Kilicoglu H. Rindflesch T. C.. (2014). Using semantic predications to uncover drug-drug interactions in clinical data. J. Biomed. Inform. 49, 134–147. 10.1016/j.jbi.2014.01.004 24448204 PMC4058371

[B136] Zhang Y. Tao C. He Y. Kanjamala P. Liu H. (2013). Network-based analysis of vaccine-related associations reveals consistent knowledge with the vaccine ontology. J. Biomed. Semantics 4:33. 10.1186/2041-1480-4-33 24209834 PMC4177205

[B137] Zhang Y. Wu H.-Y. Du J. Xu J. Wang J. Tao C. (2016). Extracting drug-enzyme relation from literature as evidence for drug drug interaction. J. Biomed. Semantics 7:11 10.1186/s13326-016-0052-6 26955465 PMC4780188

[B138] Zhichkin P. E. Athey B. D. Avigan M. I. Abernethy D. R. (2012). Needs for an expanded ontology-based classification of adverse drug reactions and related mechanisms. Clin. Pharmacol. Ther. 91, 963–965. 10.1038/clpt.2012.41 22609907

