# Supplementary material for: Computational Advances in Drug Safety: Systematic and Mapping Review of Knowledge Engineering Based Approaches
Source: Front Pharmacol. 2019 May 17;10:415. doi: 10.3389/fphar.2019.00415 (PMC6533857; doi:10.3389/fphar.2019.00415)
Supplement: Supplementary file 1 [file Table_1.DOCX]

**Bibliographic Queries**

**a) PubMed:**

("knowledge base"[tiab] OR semantic*[tiab] OR ontolog*[tiab] OR "knowledge-based"[tiab] OR "knowledge based"[tiab] OR "rule-based"[tiab] OR "rule based"[tiab] OR "knowledge engineering"[tiab] OR "linked data"[tiab] OR "linked-data"[tiab] OR reasoning[tiab] OR reasoner[tiab] OR inference[tiab] OR inferencing[tiab])

AND

("adverse drug event"[tiab] OR "adverse drug events"[tiab] OR "adverse event"[tiab] OR "adverse events"[tiab] OR "adverse drug effect"[tiab] OR "adverse drug effects"[tiab] OR ADE[tiab] OR ADR[tiab] OR "adverse effect"[tiab] OR "adverse effects"[tiab] OR "adverse effect of drug"[tiab] OR "adverse effects of drug"[tiab] OR "adverse drug reaction"[tiab] OR "adverse drug reactions"[tiab] OR "adverse reaction"[tiab] OR "adverse reactions"[tiab] OR "drug safety"[tiab] OR "drug safety risk"[tiab] OR "drug safety risks"[tiab] OR "drug interaction"[tiab] OR "drug interactions"[tiab] OR "drug-drug interaction"[tiab] OR "drug-drug interactions"[tiab] OR "drug drug interactions"[tiab] OR "drug drug interaction"[tiab] OR pharmacovigilance[tiab] OR "drug surveillance"[tiab] OR "medication safety"[tiab])

AND

("journal article"[pt] NOT Bibliography[pt] NOT Portraits[pt] NOT Comment[pt] NOT Editorial[pt] NOT Letter[pt] NOT News[pt] NOT Case Reports[pt] NOT Published Erratum[pt] NOT Historical Article[pt] NOT Legal Cases[pt] NOT legislation[pt] NOT "Yearb Med Inform"[jour] NOT "Stud Health Technol Inform"[jour] NOT Medinfo[jour] NOT "Proc Annu Symp Comput Appl Med Care"[jour] NOT "Water Sci Techno"[jour] NOT "Mayo Clin Proc"[jour] NOT "Sci Total Environ"[jour] NOT "Waste Manag"[jour] NOT "Waste Manag Res"[jour] NOT "Environ Sci Technol"[jour] NOT "Environ Manage"[jour] NOT "Conf Proc IEEE Eng Med Biol Soc"[jour] NOT "AMIA Annu Symp Proc"[jour] NOT "AMIA Jt Summits Transl Sci Proc"[jour] NOT "Pac Symp Biocomput"[jour])

AND "english"[LA]

AND hasabstract

AND 2006:2017[DP]

**b) Web of Science^[[1]](#footnote-1)^:**

((TS =("adverse drug event" OR "adverse drug events" OR "adverse event" OR "adverse events" OR "adverse drug effect" OR "adverse drug effects" OR ADE[tiab] OR ADR[tiab] OR "adverse effect" OR "adverse effects" OR "adverse effect of drug" OR "adverse effects of drug" OR "adverse drug reaction" OR "adverse drug reactions" OR "adverse reaction" OR "adverse reactions" OR "drug safety" OR "drug safety risk" OR "drug safety risks" OR "drug interaction" OR "drug interactions" OR "drug-drug interaction" OR "drug-drug interactions" OR "drug drug interactions" OR "drug drug interaction" OR pharmacovigilance OR "drug surveillance" OR "medication safety"))

AND

(TS=("knowledge base" OR semantic* OR ontolog* OR "knowledge-based" OR "knowledge based" OR "rule-based" OR "rule based" OR "knowledge engineering" OR "linked data" OR "linked-data" OR reasoning OR reasoner OR inference OR inferencing))

AND

(SU=("Medical Informatics" OR "Health Care Sciences & Services" OR "Computer Science" OR "Pharmacology & Pharmacy" OR "Mathematics" OR "Engineering") OR WC=("Medical Informatics" OR "Health Care Sciences & Services" OR "Computer Science" OR "Pharmacology & Pharmacy" OR "Mathematics" OR "Engineering"))

NOT PMID=("1*" OR "2*" OR "3*" OR "4*" OR "5*" OR "6*" OR "7*" OR "8*" OR "9*" OR "0*"))

AND LANGUAGE: (English)

AND DOCUMENT TYPES: (Article)

Indexes=SCI-EXPANDED, SSCI, A&HCI, CPCI-S, CPCI-SSH, ESCI Timespan=2006-2017

1. Remark: The query has to be submitted in parts. [↑](#footnote-ref-1)
