# Supplementary material for: Computational Advances in Drug Safety: Systematic and Mapping Review of Knowledge Engineering Based Approaches
Source: Front Pharmacol. 2019 May 17;10:415. doi: 10.3389/fphar.2019.00415 (PMC6533857; doi:10.3389/fphar.2019.00415)
Supplement: Supplementary file 3 [file Table_3.DOCX]

# Articles selected in the study

1. Abdelaziz, I., Hassanzadeh, O., Zhang, P., and Sadoghi, M. (2017). Large-scale structural and textual similarity-based mining of knowledge graph to predict drug–drug interactions. *Web Semant. Sci. Serv. Agents World Wide Web* 44, 104–117. doi:10.1016/J.WEBSEM.2017.06.002.
2. Alecu, I., Bousquet, C., and Jaulent, M.-C. (2008). A case report: using SNOMED CT for grouping Adverse Drug Reactions Terms. *BMC Med. Inform. Decis. Mak.* 8 Suppl 1, S4. doi:10.1186/1472-6947-8-S1-S4.
3. Arikuma, T., Yoshikawa, S., Azuma, R., Watanabe, K., Matsumura, K., and Konagaya, A. (2008). Drug interaction prediction using ontology-driven hypothetical assertion framework for pathway generation followed by numerical simulation. *BMC Bioinformatics* 9 Suppl 6, S11. doi:10.1186/1471-2105-9-S6-S11.
4. Audeh, B., Beigbeder, M., Zimmermann, A., Jaillon, P., and Bousquet, C. (2017). Vigi4Med Scraper: A Framework for Web Forum Structured Data Extraction and Semantic Representation. *PLoS One* 12. doi:10.1371/journal.pone.0169658.
5. Bisgin, H., Liu, Z., Fang, H., Xu, X., and Tong, W. (2011). Mining FDA drug labels using an unsupervised learning technique--topic modeling. *BMC Bioinformatics* 12 Suppl 10, S11. doi:10.1186/1471-2105-12-S10-S11.
6. Bousquet, C., Sadou, É., Souvignet, J., Jaulent, M.-C., and Declerck, G. (2014). Formalizing MedDRA to support semantic reasoning on adverse drug reaction terms. *J. Biomed. Inform.* 49, 282–291. doi:10.1016/j.jbi.2014.03.012.
7. Boyce, R. D., Horn, J. R., Hassanzadeh, O., Waard, A. de, Schneider, J., Luciano, J. S., et al. (2013). Dynamic enhancement of drug product labels to support drug safety, efficacy, and effectiveness. *J. Biomed. Semantics* 4, 5. doi:10.1186/2041-1480-4-5.
8. Boyce, R. D., Ryan, P. B., Norén, G. N., Schuemie, M. J., Reich, C., Duke, J., et al. (2014). Bridging Islands of Information to Establish an Integrated Knowledge Base of Drugs and Health Outcomes of Interest. *Drug Saf.* 37, 557–567. doi:10.1007/s40264-014-0189-0.
9. Bravo, À., Li, T. S., Su, A. I., Good, B. M., and Furlong, L. I. (2016). Combining machine learning, crowdsourcing and expert knowledge to detect chemical-induced diseases in text. *Database (Oxford).* 2016. doi:10.1093/database/baw094.
10. Cai, M.-C., Xu, Q., Pan, Y.-J., Pan, W., Ji, N., Li, Y.-B., et al. (2015). ADReCS: an ontology database for aiding standardization and hierarchical classification of adverse drug reaction terms. *Nucleic Acids Res.* 43, 907-D913. doi:10.1093/nar/gku1066.
11. Cai, R., Liu, M., Hu, Y., Melton, B. L., Matheny, M. E., Xu, H., et al. (2017). Identification of adverse drug-drug interactions through causal association rule discovery from spontaneous adverse event reports. *Artif. Intell. Med.* 76, 7–15. doi:10.1016/j.artmed.2017.01.004.
12. Cañada, A., Capella-Gutierrez, S., Rabal, O., Oyarzabal, J., Valencia, A., and Krallinger, M. (2017). LimTox: a web tool for applied text mining of adverse event and toxicity associations of compounds, drugs and genes. *Nucleic Acids Res.* 45, W484–W489. doi:10.1093/nar/gkx462.
13. Ceusters, W., Capolupo, M., de Moor, G., Devlies, J., and Smith, B. (2011). An evolutionary approach to realism-based adverse event representations. *Methods Inf. Med.* 50, 62–73. doi:10.3414/ME10-02-0016.
14. Cheng, F., Li, W., Wang, X., Zhou, Y., Wu, Z., Shen, J., et al. (2013). Adverse Drug Events: Database Construction and in Silico Prediction. *J. Chem. Inf. Model.* 53, 744–752. doi:10.1021/ci4000079.
15. Cheng, F., and Zhao, Z. (2014). Machine learning-based prediction of drug-drug interactions by integrating drug phenotypic, therapeutic, chemical, and genomic properties. *J. Am. Med. Inform. Assoc.* 21, 278–86. doi:10.1136/amiajnl-2013-002512.
16. Cocos, A., Fiks, A. G., and Masino, A. J. (2017). Deep learning for pharmacovigilance: recurrent neural network architectures for labeling adverse drug reactions in Twitter posts. *J. Am. Med. Informatics Assoc.* 24, 813–821. doi:10.1093/jamia/ocw180.
17. Cohen, T., and Widdows, D. (2017). Embedding of semantic predications. *J. Biomed. Inform.* 68, 150–166. doi:10.1016/j.jbi.2017.03.003.
18. Courtot, M., Brinkman, R. R., and Ruttenberg, A. (2014). The logic of surveillance guidelines: An analysis of vaccine adverse event reports from an ontological perspective. *PLoS One*. doi:10.1371/journal.pone.0092632.
19. Declerck, G., Hussain, S., Daniel, C., Yuksel, M., Laleci, G. B., Twagirumukiza, M., et al. (2015). Bridging Data Models and Terminologies to Support Adverse Drug Event Reporting Using EHR Data. *Methods Inf. Med.* 54, 24–31. doi:10.3414/ME13-02-0025.
20. Doulaverakis, C., Nikolaidis, G., Kleontas, A., and Kompatsiaris, I. (2014). Panacea, a semantic-enabled drug recommendations discovery framework. *J. Biomed. Semantics* 5, 13. doi:10.1186/2041-1480-5-13.
21. Dupuch, M., and Grabar, N. (2015). Semantic distance-based creation of clusters of pharmacovigilance terms and their evaluation. *J. Biomed. Inform.* 54, 174–185. doi:10.1016/J.JBI.2014.11.007.
22. Eshleman, R., and Singh, R. (2016). Leveraging graph topology and semantic context for pharmacovigilance through twitter-streams. *BMC Bioinformatics* 17, 335. doi:10.1186/s12859-016-1220-5.
23. Gottlieb, A., Stein, G. Y., Oron, Y., Ruppin, E., and Sharan, R. (2012). INDI: a computational framework for inferring drug interactions and their associated recommendations. *Mol. Syst. Biol.* 8, 592. doi:10.1038/msb.2012.26.
24. Guo, A., Racz, R., Hur, J., Lin, Y., Xiang, Z., Zhao, L., et al. (2016). Ontology-based collection, representation and analysis of drug-associated neuropathy adverse events. *J. Biomed. Semantics* 7, 29. doi:10.1186/s13326-016-0069-x.
25. Gurulingappa, H., Mateen-Rajput, A., and Toldo, L. (2012). Extraction of potential adverse drug events from medical case reports. *J. Biomed. Semantics* 3, 15. doi:10.1186/2041-1480-3-15.
26. He, L., Yang, Z., Zhao, Z., Lin, H., and Li, Y. (2013). Extracting drug-drug interaction from the biomedical literature using a stacked generalization-based approach. *PLoS One* 8, e65814. doi:10.1371/journal.pone.0065814.
27. He, Y., Sarntivijai, S., Lin, Y., Xiang, Z., Guo, A., Zhang, S., et al. (2014b). OAE: The Ontology of Adverse Events. *J. Biomed. Semantics* 5, 29. doi:10.1186/2041-1480-5-29.
28. Henegar, C., Bousquet, C., Lillo-Le Louët, A., Degoulet, P., and Jaulent, M.-C. (2006). Building an ontology of adverse drug reactions for automated signal generation in pharmacovigilance. *Comput. Biol. Med.* 36, 748–767. doi:10.1016/j.compbiomed.2005.04.009.
29. Henriksson, A., Kvist, M., Dalianis, H., and Duneld, M. (2015). Identifying adverse drug event information in clinical notes with distributional semantic representations of context. *J. Biomed. Inform.* 57, 333–349. doi:10.1016/j.jbi.2015.08.013.
30. Henriksson, A., Zhao, J., Dalianis, H., and Boström, H. (2016). Ensembles of randomized trees using diverse distributed representations of clinical events. *BMC Med. Inform. Decis. Mak.* 16 Suppl 2, 69. doi:10.1186/s12911-016-0309-0.
31. Herrero-Zazo, M., Segura-Bedmar, I., Hastings, J., and Martínez, P. (2015). DINTO: Using OWL Ontologies and SWRL Rules to Infer Drug–Drug Interactions and Their Mechanisms. *J. Chem. Inf. Model.* 55, 1698–1707. doi:10.1021/acs.jcim.5b00119.
32. Hogan, W. R., Hanna, J., Hicks, A., Amirova, S., Bramblett, B., Diller, M., et al. (2017). Therapeutic indications and other use-case-driven updates in the drug ontology: anti-malarials, anti-hypertensives, opioid analgesics, and a large term request. *J. Biomed. Semantics* 8, 10. doi:10.1186/s13326-017-0121-5.
33. Huang, L.-C., Wu, X., and Chen, J. Y. (2011). Predicting adverse side effects of drugs. *BMC Genomics* 12 Suppl 5, S11. doi:10.1186/1471-2164-12-S5-S11.
34. Hur, J., Ozgür, A., Xiang, Z., and He, Y. (2012). Identification of fever and vaccine-associated gene interaction networks using ontology-based literature mining. *J. Biomed. Semantics* 3, 18. doi:10.1186/2041-1480-3-18.
35. Iqbal, E., Mallah, R., Rhodes, D., Wu, H., Romero, A., Chang, N., et al. (2017). ADEPt, a semantically-enriched pipeline for extracting adverse drug events from free-text electronic health records. *PLoS One* 12, e0187121. doi:10.1371/journal.pone.0187121.
36. Iyer, S. V., Harpaz, R., LePendu, P., Bauer-Mehren, A., and Shah, N. H. (2014). Mining clinical text for signals of adverse drug-drug interactions. *J. Am. Med. Informatics Assoc.* 21, 353–362. doi:10.1136/amiajnl-2013-001612.
37. Jiang, G., Liu, H., Solbrig, H. R., and Chute, C. G. (2015). Mining severe drug-drug interaction adverse events using Semantic Web technologies: a case study. *BioData Min.* 8. doi:10.1186/s13040-015-0044-6.
38. Kang, N., Singh, B., Bui, C., Afzal, Z., van Mulligen, E. M., and Kors, J. A. (2014). Knowledge-based extraction of adverse drug events from biomedical text. *BMC Bioinformatics* 15, 64. doi:10.1186/1471-2105-15-64.
39. Kawazoe, Y., Imai, T., and Ohe, K. (2016). A Querying Method over RDF-ized Health Level Seven v2.5 Messages Using Life Science Knowledge Resources. *JMIR Med. Informatics* 4, e12. doi:10.2196/medinform.5275.
40. Knowledge Base workgroup of the Observational Health Data Sciences and Informatics (OHDSI) collaborative (2017). Large-scale adverse effects related to treatment evidence standardization (LAERTES): an open scalable system for linking pharmacovigilance evidence sources with clinical data. *J. Biomed. Semantics* 8, 11. doi:10.1186/s13326-017-0115-3.
41. Koutkias, V. G., and Jaulent, M.-C. (2015). Computational Approaches for Pharmacovigilance Signal Detection: Toward Integrated and Semantically-Enriched Frameworks. *Drug Saf.* 38, 219–232. doi:10.1007/s40264-015-0278-8.
42. Koutkias, V., and Jaulent, M.-C. (2016). A Multiagent System for Integrated Detection of Pharmacovigilance Signals. *J. Med. Syst.* 40, 37. doi:10.1007/s10916-015-0378-0.
43. Koutkias, V., Kilintzis, V., Stalidis, G., Lazou, K., Niès, J., Durand-Texte, L., et al. (2012). Knowledge engineering for adverse drug event prevention: On the design and development of a uniform, contextualized and sustainable knowledge-based framework. *J. Biomed. Inform.* 45, 495–506. doi:10.1016/j.jbi.2012.01.007.
44. Lamy, J.-B., Berthelot, H., Favre, M., Ugon, A., Duclos, C., and Venot, A. (2017). Using visual analytics for presenting comparative information on new drugs. *J. Biomed. Inform.* 71, 58–69. doi:10.1016/J.JBI.2017.04.019.
45. Lin, S.-F., Xiao, K.-T., Huang, Y.-T., Chiu, C.-C., and Soo, V.-W. (2010). Analysis of adverse drug reactions using drug and drug target interactions and graph-based methods. *Artif. Intell. Med.* 48, 161–166. doi:10.1016/j.artmed.2009.11.002.
46. Lin, Y., and He, Y. (2012). Ontology representation and analysis of vaccine formulation and administration and their effects on vaccine immune responses. *J. Biomed. Semantics* 3, 17. doi:10.1186/2041-1480-3-17.
47. Lin, Y., and He, Y. (2014). The ontology of genetic susceptibility factors (OGSF) and its application in modeling genetic susceptibility to vaccine adverse events. *J. Biomed. Semantics* 5, 19. doi:10.1186/2041-1480-5-19.
48. Liu, J., Zhao, S., and Wang, G. (2018). SSEL-ADE: A semi-supervised ensemble learning framework for extracting adverse drug events from social media. *Artif. Intell. Med.* 84, 34–49. doi:10.1016/J.ARTMED.2017.10.003.
49. Liu, J., Zhao, S., and Zhang, X. (2016). An ensemble method for extracting adverse drug events from social media. *Artif. Intell. Med.* 70, 62–76. doi:10.1016/J.ARTMED.2016.05.004.
50. Liu, Q., Wang, J., Zhu, Y., and He, Y. (2017). Ontology-based systematic representation and analysis of traditional Chinese drugs against rheumatism. *BMC Syst. Biol.* 11, 130. doi:10.1186/s12918-017-0510-5.
51. Liu, X., and Chen, H. (2015). A research framework for pharmacovigilance in health social media: Identification and evaluation of patient adverse drug event reports. *J. Biomed. Inform.* 58. doi:10.1016/j.jbi.2015.10.011.
52. Lowe, D. M., O’Boyle, N. M., and Sayle, R. A. (2016). Efficient chemical-disease identification and relationship extraction using Wikipedia to improve recall. *Database (Oxford).* 2016. doi:10.1093/database/baw039.
53. Marcos, E., Zhao, B., and He, Y. (2013). The Ontology of Vaccine Adverse Events (OVAE) and its usage in representing and analyzing adverse events associated with US-licensed human vaccines. *J. Biomed. Semantics* 4, 40. doi:10.1186/2041-1480-4-40.
54. Neubert, A., Dormann, H., Prokosch, H.-U., Bürkle, T., Rascher, W., Sojer, R., et al. (2013). E-pharmacovigilance: development and implementation of a computable knowledge base to identify adverse drug reactions. *Br. J. Clin. Pharmacol.* 76 Suppl 1, 69–77. doi:10.1111/bcp.12127.
55. Nguyen, T., Larsen, M. E., O’Dea, B., Phung, D., Venkatesh, S., and Christensen, H. (2017). Estimation of the prevalence of adverse drug reactions from social media. *Int. J. Med. Inform.* 102, 130–137. doi:10.1016/j.ijmedinf.2017.03.013.
56. Nikfarjam, A., Sarker, A., O’Connor, K., Ginn, R., and Gonzalez, G. (2015). Pharmacovigilance from social media: mining adverse drug reaction mentions using sequence labeling with word embedding cluster features. *J. Am. Med. Inform. Assoc.* 22, 671–81. doi:10.1093/jamia/ocu041.
57. Noor, A., Assiri, A., Ayvaz, S., Clark, C., and Dumontier, M. (2016). Drug-drug interaction discovery and demystification using Semantic Web technologies. *J. Am. Med. Informatics Assoc.*, ocw128. doi:10.1093/jamia/ocw128.
58. Personeni, G., Bresso, E., Devignes, M.-D., Dumontier, M., Smaïl-Tabbone, M., and Coulet, A. (2017). Discovering associations between adverse drug events using pattern structures and ontologies. *J. Biomed. Semantics* 8, 29. doi:10.1186/s13326-017-0137-x.
59. Piñero, J., Bravo, À., Queralt-Rosinach, N., Gutiérrez-Sacristán, A., Deu-Pons, J., Centeno, E., et al. (2017). DisGeNET: a comprehensive platform integrating information on human disease-associated genes and variants. *Nucleic Acids Res.* 45, D833–D839. doi:10.1093/nar/gkw943.
60. Sarker, A., and Gonzalez, G. (2015). Portable automatic text classification for adverse drug reaction detection via multi-corpus training. *J. Biomed. Inform.* 53, 196–207. doi:10.1016/j.jbi.2014.11.002.
61. Sarntivijai, S., Xiang, Z., Shedden, K. A., Markel, H., Omenn, G. S., Athey, B. D., et al. (2012). Ontology-Based Combinatorial Comparative Analysis of Adverse Events Associated with Killed and Live Influenza Vaccines. *PLoS One* 7, e49941. doi:10.1371/journal.pone.0049941.
62. Sarntivijai, S., Zhang, S., Jagannathan, D. G., Zaman, S., Burkhart, K. K., Omenn, G. S., et al. (2016). Linking MedDRA(®)-Coded Clinical Phenotypes to Biological Mechanisms by the Ontology of Adverse Events: A Pilot Study on Tyrosine Kinase Inhibitors. *Drug Saf.* 39, 697–707. doi:10.1007/s40264-016-0414-0.
63. Segura-Bedmar, I., Crespo, M., de Pablo-Sánchez, C., and Martínez, P. (2010). Resolving anaphoras for the extraction of drug-drug interactions in pharmacological documents. *BMC Bioinformatics* 11 Suppl 2, S1. doi:10.1186/1471-2105-11-S2-S1.
64. Segura-Bedmar, I., and Martínez, P. (2017). Simplifying drug package leaflets written in Spanish by using word embedding. *J. Biomed. Semantics* 8, 45. doi:10.1186/s13326-017-0156-7.
65. Segura-Bedmar, I., Martínez, P., and de Pablo-Sánchez, C. (2011). A linguistic rule-based approach to extract drug-drug interactions from pharmacological documents. *BMC Bioinformatics* 12 Suppl 2, S1. doi:10.1186/1471-2105-12-S2-S1.
66. Shang, N., Xu, H., Rindflesch, T. C., and Cohen, T. (2014). Identifying plausible adverse drug reactions using knowledge extracted from the literature. *J. Biomed. Inform.* 52, 293–310. doi:10.1016/j.jbi.2014.07.011.
67. Souvignet, J., Declerck, G., Asfari, H., Jaulent, M.-C., and Bousquet, C. (2016). OntoADR a semantic resource describing adverse drug reactions to support searching, coding, and information retrieval. *J. Biomed. Inform.* 63, 100–107. doi:10.1016/j.jbi.2016.06.010.
68. Tao, C., He, Y., Yang, H., Poland, G. A., and Chute, C. G. (2012). Ontology-based time information representation of vaccine adverse events in VAERS for temporal analysis. *J. Biomed. Semantics* 3, 13. doi:10.1186/2041-1480-3-13.
69. Tari, L., Anwar, S., Liang, S., Cai, J., and Baral, C. (2010). Discovering drug-drug interactions: a text-mining and reasoning approach based on properties of drug metabolism. *Bioinformatics* 26, i547-53. doi:10.1093/bioinformatics/btq382.
70. Vandervalk, B., McCarthy, E. L., Cruz-Toledo, J., Klein, A., Baker, C. J. O., Dumontier, M., et al. (2013). The SADI Personal Health Lens: A Web Browser-Based System for Identifying Personally Relevant Drug Interactions. *JMIR Res. Protoc.* 2, e14. doi:10.2196/resprot.2315.
71. Voss, E. A., Boyce, R. D., Ryan, P. B., van der Lei, J., Rijnbeek, P. R., and Schuemie, M. J. (2017). Accuracy of an automated knowledge base for identifying drug adverse reactions. *J. Biomed. Inform.* 66, 72–81. doi:10.1016/J.JBI.2016.12.005.
72. Wang, L., Jiang, G., Li, D., and Liu, H. (2014). Standardizing adverse drug event reporting data. *J. Biomed. Semantics* 5. doi:10.1186/2041-1480-5-36.
73. Wang, L., Li, M., Xie, J., Cao, Y., Liu, H., and He, Y. (2017). Ontology-based systematical representation and drug class effect analysis of package insert-reported adverse events associated with cardiovascular drugs used in China. *Nat. Sci. Reports* 7. doi:10.1038/s41598-017-12580-4.
74. Xie, J., Codd, C., Mo, K., and He, Y. (2016a). Differential Adverse Event Profiles Associated with BCG as a Preventive Tuberculosis Vaccine or Therapeutic Bladder Cancer Vaccine Identified by Comparative Ontology-Based VAERS and Literature Meta-Analysis. *PLoS One* 11, e0164792. doi:10.1371/journal.pone.0164792.
75. Xie, J., Zhao, L., Zhou, S., and He, Y. (2016b). Statistical and Ontological Analysis of Adverse Events Associated with Monovalent and Combination Vaccines against Hepatitis A and B Diseases. *Sci. Rep.* 6, 34318. doi:10.1038/srep34318.
76. Xu, R., and Wang, Q. (2013). A semi-supervised approach to extract pharmacogenomics-specific drug-gene pairs from biomedical literature for personalized medicine. *J. Biomed. Inform.* 46, 585–93. doi:10.1016/j.jbi.2013.04.001.
77. Yuksel, M., Gonul, S., Laleci Erturkmen, G. B., Sinaci, A. A., Invernizzi, P., Facchinetti, S., et al. (2016). An Interoperability Platform Enabling Reuse of Electronic Health Records for Signal Verification Studies. *Biomed Res. Int.* 2016, 6741418. doi:10.1155/2016/6741418.
78. Zhang, R., Cairelli, M. J., Fiszman, M., Rosemblat, G., Kilicoglu, H., Rindflesch, T. C., et al. (2014). Using semantic predications to uncover drug-drug interactions in clinical data. *J. Biomed. Inform.* 49, 134–47. doi:10.1016/j.jbi.2014.01.004.
79. Zhang, Y., Tao, C., He, Y., Kanjamala, P., and Liu, H. (2013). Network-based analysis of vaccine-related associations reveals consistent knowledge with the vaccine ontology. *J. Biomed. Semantics* 4, 33. doi:10.1186/2041-1480-4-33.
80. Zhang, Y., Wu, H.-Y., Du, J., Xu, J., Wang, J., Tao, C., et al. (2016). Extracting drug-enzyme relation from literature as evidence for drug drug interaction. *J. Biomed. Semantics* 7, 11. doi:10.1186/s13326-016-0052-6.
